# A greedy feature selection algorithm for Big Data of high dimensionality

**DOI:** 10.1007/s10994-018-5748-7

**Published:** 2018-08-07

**Authors:** Ioannis Tsamardinos, Giorgos Borboudakis, Pavlos Katsogridakis, Polyvios Pratikakis, Vassilis Christophides

**Affiliations:** 1 Computer Science Department, University of Crete, Heraklion, Greece; 2 Gnosis Data Analysis PC, Heraklion, Greece; 3 Institute of Computer Science, Foundation for Research and Technology - Hellas, Heraklion, Greece; 4 INRIA, Paris, France

**Keywords:** Feature selection, Variable selection, Forward selection, Big Data, Data analytics

## Abstract

We present the *Parallel, Forward–Backward with Pruning* (PFBP) algorithm for *feature selection* (FS) for Big Data of high dimensionality. PFBP partitions the data matrix both in terms of rows as well as columns. By employing the concepts of *p*-values of conditional independence tests and meta-analysis techniques, PFBP relies only on computations local to a partition while minimizing communication costs, thus massively parallelizing computations. Similar techniques for combining local computations are also employed to create the final predictive model. PFBP employs asymptotically sound heuristics to make early, approximate decisions, such as *Early Dropping* of features from consideration in subsequent iterations, *Early Stopping* of consideration of features within the same iteration, or *Early Return* of the winner in each iteration. PFBP provides asymptotic guarantees of optimality for data distributions *faithfully* representable by a causal network (Bayesian network or maximal ancestral graph). Empirical analysis confirms a super-linear speedup of the algorithm with increasing sample size, linear scalability with respect to the number of features and processing cores. An extensive comparative evaluation also demonstrates the effectiveness of PFBP against other algorithms in its class. The heuristics presented are general and could potentially be employed to other greedy-type of FS algorithms. An application on simulated Single Nucleotide Polymorphism (SNP) data with 500K samples is provided as a use case.

## Introduction

Creating predictive models from data requires sophisticated machine learning, pattern recognition, and statistical modeling techniques. When applied to Big Data settings these algorithms need to scale not only to millions of training instances (samples, examples) but also millions of predictive quantities (interchangeably called features, variables, or attributes) (Zhao et al. 2013; Zhai et al. 2014; Bolón-Canedo et al. 2015a, b). A common way to *reduce the data dimensionality* consists of selecting only a subset of the original features that retains all of the predictive information regarding an outcome of interest *T*. Specifically, the objective of the Feature Selection (FS) problem can be defined as identifying a feature subset that is of *minimal-size* and *collectively* (multivariately) *optimally predictive*^1^ w.r.t. *T*.^2^ By removing *irrelevant as well as redundant* (related to the concept of *weakly relevant*) features (John et al. 1994), FS essentially facilitates the learning task. It results in predictive models with fewer features that are easier to inspect, visualize, understand, and faster to apply. Thus, FS provides valuable intuition on the data generating mechanism and *is a primary tool for knowledge discovery; deep connections of the solutions to the FS with the causal mechanisms that generate the data have been found* (Tsamardinos and Aliferis 2003; Koller and Sahami 1996, Pearl 1988). Indeed, FS is often the primary task of an analysis, while predictive modeling is only a by-product.

Designing a FS algorithm is challenging because by definition it is a combinatorial problem; the FS problem is NP-hard even for linear regression problems (Welch 1982). An exhaustive search of all feature subsets is impractical except for quite small sized feature spaces. Heuristic search strategies and approximating assumptions are required to scale up FS, ranging from convex relaxations and parametric assumptions such as linearity [e.g., the Lasso algorithm (Tibshirani 1996)] to causally-inspired, non-parametric assumptions, such as faithfulness of the data distribution to a causal model (Pearl and Verma 1995; Spirtes et al. 2000).

Specifically, in the context of Big Data featuring both high dimensionality and/or high sample volume, computations become *CPU-intensive* as well as and *data-intensive* and cannot be handled by a single machine.^3^ The main challenges arising in this context are (a) how can data be partitioned both horizontally (over samples) and vertically (over features), called *hybrid-partitioning*, so that *computations can be performed locally in each block* and combined globally with a *minimal communication overhead*; (b) what *heuristics* can quickly (e.g., without the need to go through all samples) and safely (providing theoretical guarantees of correctness) eliminate irrelevant and redundant features. Hybrid partitioning over both data samples and learned models (Xing et al. 2016; Lee et al. 2014) is an open research issue in Big ML algorithms while safe FS heuristics has been proposed only for sparse Big Data (Singh et al. 2009; Ramrez-Gallego et al. 2017), i.e., for data where a large percentage of values are the same (typically zeros).

To address these challenges we introduce the *Parallel, Forward–Backward with Pruning* (*PFBP*) algorithm for Big Volume Data. PFBP does not rely on data sparsity and is generally applicable to both dense and sparse datasets; in the future, it could be extended to include optimizations specifically designed for sparse datasets. PFBP is based on *statistical tests of conditional independence* and it is inspired by statistical causal modeling that represents the joint probability distribution as a causal model and specifically the theory of Bayesian networks and maximal ancestral graphs (Pearl 2000; Spirtes et al. 2000; Richardson and Spirtes 2002).

To tackle parallelization with hybrid partitioning (challenge (a) above), PFBP decisions rely on *p*-values and log-likelihoods returned by the independence tests computed *locally* on each data partition; these values are then combined together using *statistical meta-analysis techniques* to produce *global* approximate *p*-values^4^ and log-likelihoods. This technique essentially minimizes PFBP’s communication cost, as only local *p*-values and log-likelihoods need to be communicated from workers to the master node in a cluster of machines at each iteration of the algorithm. The idea of combining local statistics to global statistics can also be applied to combining local predictive models trained on the currently selected features, to provide global predictive models. This type of constructing a global predictive modeled is evaluated in the experimental section in the context of logistic regression models.

To reduce the number and workload of iterations required to compute a FS solution (challenge (b) above), PFBP relies on several heuristics. First, it adapts for Big Data a heuristic called *Early Dropping* recently introduced in Borboudakis and Tsamardinos (2017). Early Dropping removes features from subsequent iterations thus significantly speeding up the algorithm. Then, PFBP is equipped with two new heuristics for *Early Stopping* of consideration of features within the same iteration, and *Early Returning* the current best feature for addition or removal. The three heuristics are implemented using *Bootstrap-based statistical tests*. They are applied on the set of currently available local *p*-values and log-likelihoods to determine whether the algorithm has seen enough samples to make safely (i.e., with high probability of correctness) early decisions.

PFBP is proven to compute the optimal feature set for distributions *faithful* (Spirtes et al. 2000) [also called stable distributions (Pearl and Verma 1995)] to a causal network represented as a Bayesian network or a maximal ancestral graph (Spirtes et al. 2000; Richardson and Spirtes 2002). These are data distributions whose set of conditional independencies coincides with the set of independencies entailed by a causal graph and the Markov Condition (Pearl and Verma 1995; Spirtes et al. 2000). Assuming faithfulness of the data distribution has led to algorithms that have been proven competitive in practice (Margaritis and Thrun 2000; Aliferis et al. 2003; Tsamardinos et al. 2003a, b; Peña et al. 2007; Aliferis et al. 2010; Lagani and Tsamardinos 2010; Lagani et al. 2013, 2017; Borboudakis and Tsamardinos 2017). We should also note that all PFBP computations are not bound to specific data-types; by supplying different conditional independence tests PFBP becomes applicable to a wide variety of data types and target variables (continuous, ordinal, nominal, right-censored time-to-event a.k.a. survival analysis, zero inflated, percentage, time-course, repeated measurements, and others; see our R package MXM (Lagani et al. 2017) for algorithms that can handle this palette of outcomes by just providing them with different conditional independence tests). Furthermore, PFBP could potentially handle data with non-linear dependencies, as long as an appropriate conditional independence test is provided [e.g., a kernel-based test (Zhang et al. 2011) or the $G^2$ test for multinomial, discrete data (Agresti 2002)].

PFBP is first evaluated on a range of simulated data to assess its scalability properties. The data are simulated from randomly generated Bayesian networks (thus, simulating a rich dependency structure) in order to incorporate a complex dependency structure among the variables and include not only irrelevant features, but also redundant features. PFBP is found to scale super-linearly with the available sample size as its heuristics allow it to make early decisions before examining all available samples. It scales linearly with the number of features and available cores. PFBP is compared on a set of real datasets spanning a range of feature and sample sizes against the main forward-selection algorithms in the literature devised for Big Data architectures. PFBP is found more computationally efficient and scalable than the state-of-the-art, selecting fewer features, without sacrificing predictive performance. The algorithm is also evaluated against several variants of information-theoretic feature selection algorithms. The latter are specialized for discrete and sparse data. PFBP is less computational efficient in this case, as it is not customized for discrete data, but exhibits a higher predictive performance. In all tasks in the evaluation, the predictive performance is assessed with models constructed with the standard logistic regression model in MLlib of the Spark distribution (hereafter SparkLR), as well as the combined predictive model constructed from the local logistic regressions ones, as proposed above (hereafter, CombLR). The experiments suggest that in several cases SparkLR fails to converge and one obtains a model that is significantly worse than random guessing (e.g., 45% accuracy of SparkLR versus 85% accuracy for random guessing). In contrast, CombLR is never worse than the trivial model more than 0.02%. While the focus of the paper is on FS, these results indicate that methods for combining local statistics and models may serve a more general purpose in Big Data analytics.

The paper is organized as follows. In Sect. 2 we provide a brief introduction to the basic concepts required to introduce our FS algorithm. The PFBP algorithm is introduced in Sect. 3. In Sect. 4 we explain the heuristics used by PFBP in detail, and show to how to implement them using bootstrap-based tests. Guidelines for setting the hyper-parameter values for the data partitioning used by PFBP are presented in Sect. 5. In Sect. 6 we list some implementation details of PFBP, which are required for a fast and robust implementation. The theoretical properties of PFBP are presented in Sect. 7. A high-level theoretical comparison of PFBP to alternative feature selection algorithms, as well as an overview of feature selection methods for Big Data is given in Sect. 8. Finally, in Sect. 9 we evaluate PFBP on synthetic data, and compare it to alternative forward-selection algorithms on 13 binary classification datasets.

## Background and preliminaries

In this section, we provide the basic notation used throughout the paper, and present the core algorithmic and statistical reasoning techniques exploited by the proposed FS algorithm. Random variables are denoted using upper-case letters (e.g. X), while sets of random variables are denoted using bold upper-case letters (e.g. $\mathbf {Z}$). We use $|\mathbf {Z}|$ to refer to the number of variables in $\mathbf {Z}$. The outcome (or target) variable will be denoted as *T*. A summary of acronyms, terms and notation is given in Table 1.

**Table 1:** Table containing common acronyms, terms and mathematical notation (left) used throughout the paper with a short description (right)

FBS	Forward–Backward Selection
PFBP	Parallel Forward–Backward with Pruning
UFS	Univariate Feature Selection
SFO	Single Feature Optimization
ED	Early Dropping
ES	Early Stopping
ER	Early Return
CombLR	Logistic regression model obtained by combining local logistic models
SparkLR	Logistic regression model obtained using Spark MLLib (Meng et al. 2016)
Iteration	Forward (backward) iteration of PFBP
Phase	Forward (backward) loop of PFBP
Run	Execution of a forward and a backward Phase by PFBP
Feature Subset	Subset of features
Sample Subset	Subset of samples
Data Block	Contains samples of one Sample Subset and one Feature Subset
Group Sample	Set of Sample Subsets
Group	Set of Data Blocks corresponding to Sample Subsets in a Group Sample
*X*	Random variable
$\mathbf {X}$	Set of random variables
$|\mathbf {X}|$	Number of elements in $\mathbf {X}$
*T*	Outcome (or target) variable
${\textsc {Pvalue}}(T,X|\mathbf {S})$	*p*-value of the conditional indendence test of *T* with *X* given $\mathbf {S}$
${\mathbf {X}}\bot {\mathbf {Y}}\,|\,{\mathbf {Z}}$	Conditional independence of $\mathbf {X}$ and $\mathbf {Y}$ given $\mathbf {Z}$
$\mathbf {X} {\not \perp } \mathbf {Y} \ | \ \mathbf {Z}$	Conditional dependence of $\mathbf {X}$ and $\mathbf {Y}$ given $\mathbf {Z}$
df	Degrees of Freedom
$\alpha$	Significance level (threshold for conditional independence tests)
$\mathcal {D}$	Dataset-2-D matrix
$\mathbf {F}$	Features in $\mathcal {D}$
$F_j$	*j*th Feature Subset
${ {nf}}$	Number of Feature Subsets
*f*	Number of features in each Feature Subset
$S_i$	*i*th Sample Subset
${ {ns}}$	Number of Sample Subsets
*s*	Number of samples in each Sample Subset
$G_q$	*q*th Group Sample
*Q*	Number of Group Samples
*C*	Number of Sample Subsets per Group Sample
$D_{i,j}$	Data Block with rows $S_i$ and columns $F_j$
$\varPi$	2-D matrix with local log *p*-values
$\pi _{i,j}$	Local *p*-value of *j*th alive variable in $\varPi$ computed on rows in $S_i$
$\pi$	Vector with combined log *p*-values
$\pi _i$	Combined log *p*-value for the *i*th alive variable
$\mathbf {S}$	Set of Selected features
$\mathbf {R}$	Set of Remaining features
$\mathbf {A}$	Set of Alive features
*B*	Number of bootstrap iterations used by bootstrap tests
$^b$	Value corresponding to *b*th bootstrap sample
$P_{ drop}$	Threshold used by bootstrap test for Early Dropping
$P_{ stop}$	Threshold used by bootstrap test for Early Stopping
$P_{ return}$	Threshold used by bootstrap test for Early Return
*lt*	Tolerance level used by bootstrap test for Early Return

### Forward–Backward feature selection

The Forward–Backward Selection algorithm (FBS) is an instance of the stepwise feature selection algorithm family (Kutner et al. 2004; Weisberg 2005). It is also one of the first and most popular algorithms for causal feature selection (Margaritis and Thrun 2000; Tsamardinos et al. 2003b). In each forward *Iteration*, FBS selects the feature that provides the largest increase in terms of predictive performance for *T*, and adds it to the set of selected variables, denoted with $\mathbf {S}$ hereon, starting from the empty set. The forward *Phase* ends when no feature further improves performance or a maximum number of selected features has been reached. In each Iteration of the backward Phase, the feature that most confidently does not reduce performance is removed from $\mathbf {S}$. The backward Phase stops when no feature can be removed without reducing performance. We use the terms *Phase* to refer to the forward and backward loops of the algorithm and *Iteration* to the part that decides which feature to add or remove next.

To determine whether predictive performance is increased or decreased when a single feature is added or removed in a greedy fashion, FBS uses conditional independence tests.^5^ An important advantage of methods relying on conditional independence tests is that it *allows one to adapt and apply the algorithm to any type of outcome* for which an appropriate statistical test of conditional independence exists. This way, the same feature selection algorithm can deal with different data types.^6^

Conditional independence of *X* with *T* given $\mathbf {S}$ implies that $P(T | \mathbf {S}, X) = P(T | \mathbf {S})$, whenever $P(\mathbf {S}) > 0$ ($\mathbf {S}$ is allowed to be the empty set). Thus, when conditional independence holds, *X* is not predictive of *T* when $\mathbf {S}$ (and only $\mathbf {S}$) is known. A conditional independence test assumes the null hypothesis that feature *X* is probabilistically independent of *T* (i.e., redundant) given a set of variables $\mathbf {S}$. The test returns a *p*-value, which corresponds to the probability that one obtains deviations from what is expected under the null hypothesis as extreme or more extreme than the deviation actually observed with the given data. When the *p*-value is low, the null hypothesis can be safely rejected: the value of *Xdoes provide predictive information* for *T* when the values of **S** are known. In practice, decisions are made using a threshold $\alpha$ (significance level) on the *p*-values; the null hypothesis is rejected if the *p*-value is below $\alpha$.
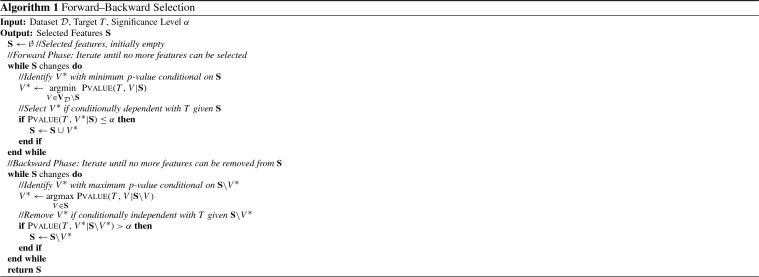


In the context of feature selection, the *p*-values returned by statistical hypotheses tests of conditional independence are employed not only to reject or accept hypotheses, but also *to rank the features according to the predictive information they provide for T* given $\mathbf {S}$. Intuitively, this can be justified by the fact that everything else being equal (i.e., sample size, type of test) the *p*-values of such tests in case of dependence have (on average) the reverse ordering with the conditional association of the variables with *T* given $\mathbf {S}$. So, the basic variant of the algorithm selects to add (remove) the feature with the lower (higher) *p*-value in each Forward (Backward) Iteration. The Forward–Backward Selection algorithm using conditional independence tests is summarized in Algorithm 1. We use $V_\mathcal {D}$ to denote the set of variables contained in dataset $\mathcal {D}$ (excluding the target *T*). The ${\textsc {Pvalue}}(T,X|\mathbf {S})$ function performs a conditional independence test of *T* and *X* given $\mathbf {S}$ and returns a *p*-value.

### Implementing independence tests using the likelihood ratio technique

There are several methods for assessing conditional independence. Examples include likelihood-ratio based tests (Wilks 1938) [or asymptotically equivalent approximations thereof like score tests and Wald tests (Engle 1984)] or kernel-based tests (Zhang et al. 2011). We focus on likelihood-ratio based tests hereafter, mostly because they are general and can be applied for different data types, although the main algorithm is not limited to such tests but can be applied with any type of test.

To construct a likelihood-ratio test for conditional independence of *T* with *X* given $\mathbf {S}$ one needs a statistical model that maximizes the log-likelihood of the data $\textit{LL}(D; \theta ) \equiv \log P(D | \theta )$ over a set of parameters $\theta$. Without loss of generality, we assume hereafter *T* is binary and consider the binary logistic regression model. For the logistic regression, the parameters $\theta$ are weight coefficients for each feature in the model and an intercept term. Subsequently, two statistical models have to be created for *T*: (i) model $M_0$ using only variables $\mathbf {S}$, and (ii) model $M_1$ using $\mathbf {S}$ and *X* resulting in corresponding log-likelihoods $\textit{LL}_0$ and $\textit{LL}_1$. The null hypothesis of independence now becomes equivalent to the hypothesis that both log-likelihoods are equal asymptotically. The test statistic function *D* of the test is defined as

$$\begin{aligned} D \equiv -\,2\times (\textit{LL}_0 - \textit{LL}_1) \end{aligned}$$

Notice that, the difference in the logs of the likelihoods corresponds to the ratio of the likelihoods, hence the name likelihood-ratio test. The test statistic is known to follow asymptotically a $\chi ^2$ distribution with $P_1 - P_0$ degrees of freedom (Wilks 1938), where $P_1$ and $P_0$ are degrees of freedom of models $M_1$ and $M_0$ respectively.^7^ When *X* is a continuous feature, only one more parameter is added to $\theta$ so the difference in degrees of freedom is 1 for this case. Categorical predictors can be used by simply encoding them as $K-1$ dummy binary features, where *K* is the number of possible values of the original feature. In this case, the difference in degrees of freedom is $K-1$. Knowing the theoretical distribution of the statistic allows one to compute the *p*-value of the test: $p = 1 - cdf (D, df)$, where $cdf$ is the cumulative probability distribution function of the $\chi ^2$ distribution with degrees of freedom *df* and *D* the observed statistic. Likelihood-ratio tests can be constructed for any type of data for which an algorithm for maximizing the data likelihood exists, such as binary, multinomial or ordinal logistic regression, linear regression and Cox regression to name a few.

Likelihood-ratio tests are *approximate* in the sense that the test statistic has a $\chi ^2$ distribution only *asymptotically*. When sample size is low, the asymptotic approximation may return inaccurate *p*-values. Thus, *to apply approximate tests it is important to ensure a sufficient number of samples is available*. This issue is treated in detail in the context of PBFP and the logistic test in “Appendix A”. Note that, the aforementioned models and the corresponding independence tests are only suited for identifying linear dependencies; certain types of non-linear dependencies may also be identifiable if one also includes interaction terms and feature transformations in the models.

### Combining *p*-values using meta-analysis techniques

A set of *p*-values stemming from testing the *same* null hypothesis (e.g. testing the conditional independence of *X* and *Y* given $\mathbf {Z}$) can be combined using statistical meta-analysis techniques into a single *p*-value. Multiple such methods exist in the literature (Loughin 2004). Fisher’s combined probability test (Fisher 1932) is one such method that has been shown to work well across many cases (Loughin 2004). It assumes that the *p*-values are independent and combines them into a single statistic using the formula

$$\begin{aligned} \text {Statistic} \equiv -2\sum _{i = 1}^{K} \log (p_i) \end{aligned}$$

where *K* is the number of *p*-values, $p_i$ is the *i*th *p*-value, and $\log$ is the natural logarithm. The statistic is then distributed as a $\chi ^2$ random variable with $2\cdot K$ degrees of freedom, from which a combined *p*-value is computed.

### Bootstrap-based hypothesis testing

The bootstrap procedure (Efron and Tibshirani 1994) can be used to compute the distribution of a statistic of interest. Bootstrapping is employed in the PFBP algorithm for making early, probabilistic decisions. Bootstrapping is a general-purpose non-parametric resampling-based procedure which works as follows: (a) resample with replacement from the input values a sample of equal size, (b) compute the statistic of interest on the bootstrap sample, (c) repeat steps (a) and (b) many times to get an estimate of the bootstrap distribution of the statistic. The bootstrap distribution can then be used to compute properties of the distribution such as confidence intervals, or to compute some condition of the statistic. A simple example application on the latter follows; more examples can be found in Efron and Tibshirani (1994).

Let $\mu _X$ denote the mean of random variable *X* and let $\hat{\mu }_X$ denote the estimate of the mean of *X* given a sample of *X*. Assume we are given a sample of size *n* of random variable *X* and we want to compute the probability that the mean of *X* is larger than 10, $P(\mu _X > 10)$. That probability is a Bernoulli random variable, and the statistic in this case is a binary valued variable (i.e., taking a value of 0 or 1 with probability $P(\mu _X > 10)$). Using bootstrapping, $P(\mu _X > 10)$ can be estimated as follows: (a) sample with replacement *n* values of *X* and create the *b*th bootstrap sample $X^b$, (b) estimate the mean of $X^b$, denoted as $\hat{\mu }^b_X$, and compute $I(\hat{\mu }^b_X > 10)$, where *I* is the indicator function returning 1 if the inequality holds and 0 otherwise, and (c) repeat (a) and (b) *B* times (e.g. $B = 1000$). $P(\mu _X > 10)$ is then computed as

$$\begin{aligned} P(\mu _X> 10) = \frac{I(\hat{\mu }_X> 10) + \sum _{i = 1}^{B} I(\hat{\mu }_X^b > 10)}{B+1} \end{aligned}$$

Note that, we also compute the statistic on the original sample (which is sample from the bootstrap distribution), and thus divide by $B+1$.^8^

### Probabilistic graphical models and Markov blankets

In this section, we give a brief overview of Bayesian networks and maximal ancestral graphs, which will be used later on to present the theoretical properties of the proposed algorithm. A more extensive exposition and rigorous treatment can be found in Spirtes et al. (2000), Richardson and Spirtes (2002) and Aliferis et al. (2010).

#### Bayesian networks

A Bayesian network $B = \langle G, P \rangle$ consists of a directed acyclic graph *G* over a set of vertices *V* and a joint distribution *P*, over random variables that correspond one-to-one to vertices in *V* (thus, no distinction is made between variables and vertices). The *Markov condition* has to hold between *G* and *P*: every variable *X* is conditionally independent of its non-descendants in *G*, given its parents, denoted by *Pa*(*X*). The Markov condition leads to a factorization of the joint probability $P(V) = \prod _i P(X_i | Pa(X_i))$. Those are not all the independencies that hold in the distribution: the Markov condition (along with the other probability axioms) implies some additional conditional independencies. A Bayesian network is called *faithful* if all and only the conditional independencies in *P* are entailed by the Markov condition. Conceptually, this faithfulness condition means that all independencies in the distribution of the data are determined by the structure of the graph *G* and not the actual parameterization of the distribution. A distribution *P* is called *faithful* (to a Bayesian network) if there is a graph *G* such that $B = \langle G, P \rangle$ is faithful. Under the Markov and faithfulness assumptions, a graphical criterion called *d-separation* (Verma and Pearl 1988; Pearl 1988) can be used to read off dependencies and independencies encoded in a Bayesian network. To define *d*-separation the notion of *colliders* is used, which are triplets of variables $\langle X, Y, Z\rangle$ with *X* and *Z* having directed edges into *Y*. Two variables *X* and *Y* are *d*-connected by a set of variables $\mathbf {Z}$ if and only if there exists a (not necessarily directed) path *p* between *X* and *Y* such that (i) for each collider *V* on *p*, *V* is either in $\mathbf {Z}$ or some descendant of *V* is in $\mathbf {Z}$, and (ii) no non-collider on *p* is in $\mathbf {Z}$. In case no such path exists, *X* and *Y* are *d*-separated given $\mathbf {Z}$. Thus, the Markov and faithfulness conditions imply that if two variables *X* and *Y* are *d*-separated (*d*-connected) given $\mathbf {Z}$, then they are conditional independent (dependent) given $\mathbf {Z}$.

#### Maximal ancestral graphs

A distribution class strictly larger than the set of faithful distributions to Bayesian networks, is the set of distributions that are marginals of faithful distributions. Unfortunately, marginals of faithful distributions are not always faithful to some Bayesian network. Thus, marginalization over some variables loses the faithfulness property: the marginal distribution cannot always be faithfully represented by a Bayesian network. However, marginals of faithful distributions can be represented by another type of graph called *directed maximal ancestral graph* (Richardson and Spirtes 2002) or DMAG. DMAGs include not only directional edges, but also bi-directional edges. DMAGs are extensions of Bayesian networks for marginal distributions and are closed under marginalization. The representation of a marginal of a faithful (to a Bayesian network) distribution is again faithful (this time to a maximal ancestral graph though, not necessarily a Bayesian Network) in the sense that all and only the conditional independencies in the distribution are implied by the Markov condition. The set of conditional independencies entailed by a DMAG is provided by a criterion similar to *d*-separation, now called *m*-separation.

#### Markov blankets in probabilistic graphical models

**Fig. 1:**
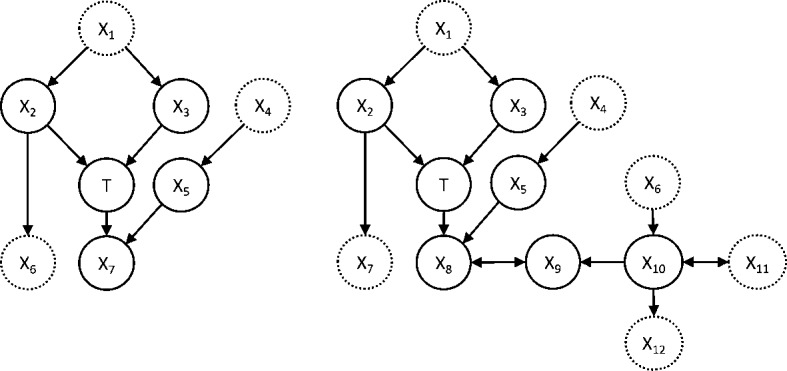
Example of Markov blankets of *T* in a Bayesian network (left) and a maximal ancestral graph (right). The nodes in the Markov blanket are shown with solid lines, and the remaining ones with dashed lines. In both cases, the Markov blanket contains all adjacent nodes (parents and children) and $X_5$ (spouse of *T*). In addition, in the maximal ancestral graph $X_9$ and $X_{10}$ are also contained, as they are connected with *T* through a collider path ($T \rightarrow X_8 \leftrightarrow X_9 \leftarrow X_{10}$)

A *Markov blanket* of *T* with respect to a set of variables $\mathbf {V}$ is defined as a minimal set $\mathbf {S}$ such that $\mathbf {V} {\setminus } \mathbf {S} {\perp } T \ | \ \mathbf {S}$, where $\mathbf {X} {\perp } T \ | \ \mathbf {S}$ denotes the conditional independence of $\mathbf {X}$ with *T* given $\mathbf {S}$. Thus, a Markov blanket of *T* is any minimal set that renders all other variables conditionally independent. An important theorem connects the Markov blanket of *T* with the feature selection problem for *T*: *under broad conditions* (Margaritis and Thrun 2000; Tsamardinos and Aliferis 2003) *a Markov blanket ofTis a solution to the feature selection problem forT*. When the distribution is faithful to a Bayesian network or DMAG, the Markov blanket of *T* is unique.^9^ In other words, *for faithful distributions, the Markov Blanket ofThas a direct graphical interpretation. The Markov blanket consists of all vertices adjacent toT*, *and all vertices that are reachable fromTthrough a collider path, which is a path where all vertices except the start and end vertices are colliders* (Borboudakis and Tsamardinos 2017). For Bayesian networks, this corresponds to the set of parents (vertices with an edge to *T*), children (vertices with an edge from *T*), and spouses (parents of children) of *T* in *G*. An example of the Markov blanket of *T* in a Bayesian network and a maximal ancestral graph are shown in Fig. 1.

## Massively parallel Forward–Backward algorithm

We provide an overview of our algorithm, called Parallel, Forward–Backward with Pruning (*PFBP*), an extension of the basic Forward–Backward Selection (FBS) algorithm (see Sect. 2.1 for a description). We will use the terminology introduced for FBS: a forward (backward) *Phase* refers to the forward (backward) loops of FBS, and an *Iteration* refers to each loop iteration that decides which variable to select (remove) next. PFBP is presented in “evolutionary” steps where successive enhancements are introduced in order to make computations local or reduce computations and communication costs; the complete algorithm is presented in Sect. 3.4. To evaluate predictive performance of candidate features we use the *p*-values of conditional independence tests, as described in Sect. 2.1. We assume the data are provided in the form of a 2-dimensional matrix $\mathcal {D}$ where rows correspond to training instances (samples) and columns to features (variables), and one of the variables is the target variable *T*. Physically, the data matrix is partitioned in sub-matrices $D_{i,j}$ and stored in a distributed fashion in *workers* in a cluster running Spark (Zaharia et al. 2010) or similar platform. Workers perform in parallel local computations on each $D_{i,j}$ and a *master* node performs the centralized, global computations.

**Fig. 2:**
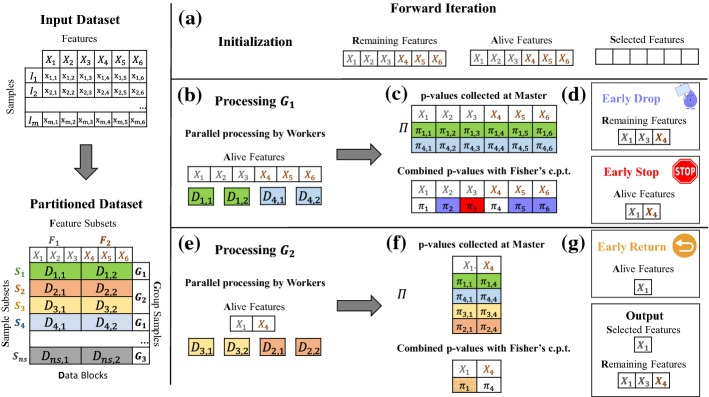
Left: Data partitioning of the algorithm. In the top the initial data matrix $\mathcal {D}$ is shown with 6 features and instances $I_1, \ldots , I_m$. In the bottom, the 6 features are partitioned to Feature Subsets $F_1 = \{1, 2, 3\}$ and $F_2 = \{4, 5, 6\}$. The rows are randomly partitioned to Sample Subsets $S_1, \ldots , S_{ ns }$, and the Sample Subsets are assigned to Group Samples. Each Block $D_{i,j}$ is physically stored as a unit. Right: Example of trace of a Forward Iteration of PFBP. **a** The $\mathbf {R}$emaining features, $\mathbf {A}$live features, and $\mathbf {S}$elected features are initialized. **b** All Data Blocks $D_{1,1}, D_{1,2}, D_{4,1}, D_{4,2}$ in the first Group are processed in parallel (by workers). **c** The resulting local *p*-values are collected (reduced) in a master node for each Alive feature and Sample Set in the first Group (as well as the likelihoods, not shown in the figure). **d** Bootstrap-based tests determine which features to Early Drop or Stop based on $\varPi$, or whether to Early Return (based on $\varLambda$, not shown in the figure). The sets $\mathbf {R}$ and $\mathbf {A}$ are updated accordingly. In this example, $X_2$, $X_5$ and $X_6$ are Dropped, $X_3$ is stopped, and only $X_1$ and $X_4$ remain Alive. Notice that always $\mathbf {A} \subseteq \mathbf {R}$. **e** The second Group is processed in parallel (by workers) containing Blocks $D_{3,1}, D_{3,2}, D_{2,1}, D_{2,2}$. **f** New local *p*-values for all features still Alive are appended to $\varPi$. If $G_2$ was the last Group, global *p*-values for the Alive features would be computed and the one with the minimum value (in this example $X_1$) would be selected for inclusion in **S**. **g** In case, $X_1$ and $X_4$ are deemed almost equally predictive (based on their log-likelihoods) the current best is Early Returned

### Data partitions in blocks and groups and parallelization strategy

We now describe the way $\mathcal {D}$ is partitioned in sub-matrices to enable parallel computations. First, the set of available features (columns) $\mathbf {F}$ is partitioned to about equal-sized *Feature Subsets*$\{F_1, \ldots , F_ nf \}$. Similarly, the samples (rows) are randomly partitioned to about equal-sized *Sample Subsets*$\{S_1, \ldots , S_ ns \}$. The row and column partitioning defines sub-matrices called *Data Blocks*$D_{i,j}$ with rows $S_i$ and features $F_j$. Sample Subsets are assigned to *QGroup Samples*$\{G_q\}_1^C$ of size *C* each, where each group sample $G_q$ is a set $\{S_{q_1}, \ldots , S_{q_n}\}$ (i.e., the set of Sample Subsets is partitioned). The Data Blocks $D_{i,j}$ with samples within a group sample $S_i \in G_q$ belong in the same *Group*. This second, higher level of grouping is required by the bootstrap tests explained in Sect. 4. Data Blocks in the same Group are processed in parallel in different workers (provided enough are available). However, Groups are processed sequentially, i.e., computation in all Blocks within a Group has to complete to begin computations in the Blocks of the next Group. Obviously, if workers are more than the Data Blocks, there is no need for defining Groups. The data partitioning scheme is shown in Fig. 2: Left. Details of how the number of Sample Sets $ns$, the number of Feature Subsets $nf$, and the number *C* of Group Samples are determined are provided in Sect. 5.

### Approximating global *p*-values by combining local *p*-values using meta-analysis

Recall that Forward–Backward Selection uses *p*-values stemming from conditional independence tests to rank the variables and to select the best one for inclusion (forward Phase) or exclusion (backward Phase). Extending the conditional independence tests to be computed over multiple Data Blocks is not straightforward, and may be computationally inefficient. For conditional independence tests based on regression models (e.g. logistic or Cox regression), a maximum-likelihood estimation over all samples has to be performed, which typically does not have a closed-form solution and thus requires the use of an iterative procedure (e.g. Newton descent). Due to its iterative nature, it results in a high communication cost rendering it computationally inefficient, especially for feature selection purposes on Big Data where numerous models have to be fit at each Iteration.

Instead of fitting full (global) regression models, we propose to perform the conditional independence tests locally on each data block, and to combine the resulting *p*-values using statistical meta-analysis techniques. Specifically, the algorithm computes *localp*-*values denoted by*$\pi _{i,k}$ for candidate feature $X_k$ from only the rows in $S_i$ of a data block $D_{i,j}$, where $F_j$ contains the feature $X_k$. This enables massive parallelization of the algorithm, as each data block can be processed independently and in parallel by a different worker (Fig. 2b). The local *p*-values $\pi _{i,k}$ are then *communicated* to the master node of the cluster, and are stored in a matrix $\varPi$ (Fig. 2c); we will use $\pi _{i,k}$ to refer to the elements of matrix $\varPi$, corresponding to the local *p*-value of $X_k$ computed on a data block containing samples in sample set $S_i$. Using the *p*-values in matrix $\varPi$, the master node combines the *p*-values to *globalp*-*values* for each feature $X_k$ using Fisher’s combined probability test (Fisher 1932) (Fig. 2c).^10^ Finally, we note that this approach is not limited to regression-based tests, but can be used with any type of conditional independence test, and is most appropriate for tests which are hard to parallelize, or computationally expensive [e.g. kernel-based tests (Zhang et al. 2011)].

Using Fisher’s combined probability test to combine local *p*-values does not necessarily lead to the same *p*-value as the one computed over all samples. There are no guarantees how close those *p*-values will be in case the null hypothesis of conditional independence holds, except that they are uniformly distributed between 0 and 1. In case the null hypothesis does not hold however (the dependency holds), one expects to reject the null hypothesis using either method in the sample limit. What is important for PFBP is to make the same decision at each Iteration, that is, that the top ranked variable given by either *p*-value computation method is the same. However, even if the top ranked variable is not the same one, PFBP may still perform well, as long as some other informative variable is ranked first. In “Appendix A” we investigate in experiments on synthetic data how both approaches compare when the task is to select the best variable at a given Iteration. We show that, if the sample size per data block is sufficiently large, combined *p*-values and *p*-values obtained from tests on all samples lead to the same choice with high probability.

For the computation of the local *p*-values on $D_{i,j}$, samples $S_i$ of the selected features $\mathbf {S}$ are required, and thus the data need to be broadcast to every worker processing $D_{i,j}$ whenever $\mathbf {S}$ is augmented, i.e., in the end of each Forward Iteration. In total, the communication cost of the algorithm is due to the assembly of all local *p*-values $\pi _{i,k}$ to determine the next feature to include (exclude), as well as the broadcast of the data for the newly added feature in $\mathbf {S}$ at the end of each forward Iteration. *We would like to emphasize that the bulk of computation of the algorithm is the calculation of localp*-*values that require expensive statistical tests and it takes place in the workers in parallel. The central computations in the master are minimal*.

### Speeding-up PFBP using pruning heuristics

In this section, we present 3 pruning heuristics used by PFBP to speed-up computation. Implementation details of the heuristics using locally computed *p*-values are presented in Sect. 4.

#### Early Dropping of features from subsequent iterations

The first addition to PFBP is the *Early Dropping* (ED) heuristic, first introduced in Borboudakis and Tsamardinos (2017) for a non-parallel version of Forward–Backward Selection. Let $\mathbf {R}$ denote the set of remaining features, that is, the set of features still under consideration for selection. Initially, $\mathbf {R} = \mathbf {F} {\setminus } \mathbf {S}$, where $\mathbf {F}$ is the set of all available features and $\mathbf {S}$ is the set of selected features, which is initially empty. At each forward Iteration, ED removes from $\mathbf {R}$ all features that are conditionally independent of the target *T* given the set of currently selected features $\mathbf {S}$. Typically, just after the first few Iterations of PFBP, only a very small proportion of the features will still remain in $\mathbf {R}$, leading to orders of magnitude of efficiency improvements even in the non-parallel version of the algorithm (Borboudakis and Tsamardinos 2017). When the set of variables $\mathbf {R}$ becomes empty, we say that PFBP finished one $\textit{Run}$. Unfortunately, the Early Dropping heuristic without further adjustments may miss important features which seem uninformative at first, but provide information for *T* when considered with features selected in subsequent Iterations. Features should be given additional opportunities to be selected by performing more Runs. Each additional Run calls the forward phase again but starts with the previously selected variables $\mathbf {S}$ and re-initializes the remaining variables to $\mathbf {R} = \mathbf {F} {\setminus } \mathbf {S}$. By default, PFBP uses 2 Runs, although a different number of Runs may be used. Typically a value of 1 or 2 is sufficient in practice, with larger values requiring more computational time while also giving stronger theoretical guarantees; the theoretical properties of PFBP with ED are described in Sect. 7 in detail; in short, assuming no statistical errors in the conditional independence tests, PFBP with 2 runs returns the Markov Blanket of *T* is distributions faithful to a Bayesian network. *Overall, by discarding variables at each Iteration, the Early Dropping heuristic allows the algorithm to scale with respect to the number of features.*

#### Early Stopping of features within the same iteration

The next addition to the algorithm regards *Early Stopping* (ES) of consideration of features *within the same* Iteration, i.e., in order to select the next best feature to select in a forward Iteration or to remove in a backward Iteration. To implement ES we introduce the set $\mathbf {A}$ of features still *Alive* (i.e., under consideration) in the current Iteration, initialized to $\mathbf {A} = \mathbf {R}$ at the beginning of each Iteration (see Fig. 2a). As the master node gathers local *p*-values for a feature $X_k$ from several Data Blocks, it may be able to determine that no more local *p*-values need to be computed for $X_k$. This is the case if these *p*-values are enough to safely decide that with high probability $X_k$ is not going to be selected for inclusion (Forward Phase) or exclusion (Backward Phase) in this Iteration (see Sect. 4 for a bootstrap-based procedure that performs this test). In this case, $X_k$ is removed from the set of alive features $\mathbf {A}$, and is not further considered in the current Iteration (see Fig. 2d). *This allows PFBP to quickly filter out variables which will not be selected at the current Iteration. Thus, ES leads to a super-linear speed-up of the feature selection algorithm with respect to the sample size: even if the sample size is doubled, the same features will be Early Stopped;p*-*values will not be computed for these features on the extra samples*.

#### Early Return of the winning feature

The final heuristic of the algorithm is called *Early Return* (ER). Recall that Early Dropping will remove features conditionally independent of *T* given $\mathbf {S}$ from this and subsequent Iterations while Early Stopping will remove non-winners from the current Iteration. However, even using both heuristics, the algorithm will keep producing local *p*-values for features $X_j$ and $X_k$ that are candidates for selection and at the same time are informationally indistinguishable (equally predictive given $\mathbf {S}$) with regards to *T* (this is the case when the residuals of $X_j$ and $X_k$ given $\mathbf {S}$ are almost collinear). When two or more features are both candidates for selection and almost indistinguishable, it does not make sense to go through the remaining data: all choices are almost equally good. Hence, Early Return terminates the *computation* in the current Iteration and returns the current best feature $X_j$, if with high probability it is not going to be much worse than the best feature at the end of the Iteration (see Fig. 2g). Again, the result is that computation in the current Iteration may not process all Groups. The motivation behind Early Return is similar to Early Stopping, in that it tries to quickly determine the next feature to select. The difference is that, Early Return tries to quickly determine whether a variable is “good enough” to be selected, in contrast to Early Stopping which discards unpromising variables.

A technical detail is that judging whether two features $X_i$ and $X_j$ are “equally predictive” is implemented using the log-likelihoods $\lambda _i$ and $\lambda _j$ of the models with predictors $\mathbf {S} \cup \{X_i\}$ and $\mathbf {S} \cup \{X_j\}$ instead of the corresponding *p*-values. The likelihoods are part of the computation of the *p*-values, thus incur no additional computational overhead.

### The parallel Forward–Backward with pruning algorithm


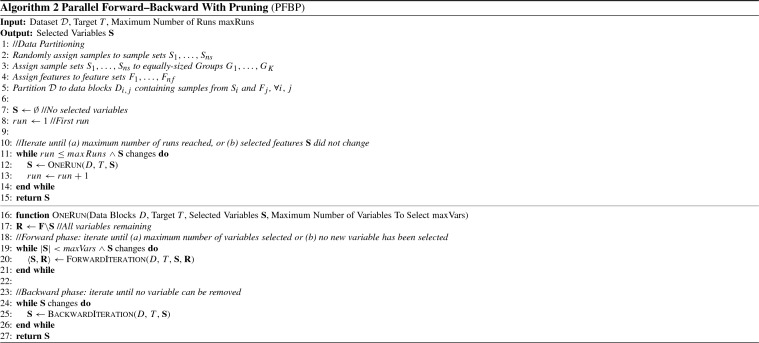


We present the proposed Parallel Forward–Backward with Pruning (PFBP) algorithm, shown in Algorithm 2. To improve readability, several arguments are omitted from function calls. PFBP takes as input a dataset $\mathcal {D}$ and the target variable of interest *T*. Initially the number of Sample Sets $ns$ and number of Feature Sets $nf$ are determined as described in Sect. 5. Then, (a) the samples are randomly assigned to Sample Sets $S_1, \dots , S_{ ns }$, to avoid any systematic biases (see also Sect. 6.1), (b) the Sample Sets $S_1, \dots , S_{ ns }$ are assigned to *Q* approximately equal-sized Groups, $G_1, \dots , G_Q$, (c) the features are assigned to feature sets $F_1, \dots , F_{ nf }$, in order of occurrence in the dataset, and (d) the dataset $\mathcal {D}$ is partitioned into data blocks $D_{i,j}$, with each such block containing samples and features corresponding to sample set $S_i$ and feature set $F_j$ respectively. The selected variables $\mathbf {S}$ are initialized to the empty set. The main loop of the algorithm performs up to $maxRuns$ Runs, as long as the selected variables $\mathbf {S}$ change. Each such Run executes a forward and a backward Phase.

The ${\textsc {OneRun}}$ function takes as input a set of data blocks *D*, the target variable *T*, a set of selected variables $\mathbf {S}$, and a limit on the number of variables to select $maxVars$. It initializes the set of remaining variables $\mathbf {R}$ to all non-selected variables $\mathbf {F} {\setminus } \mathbf {S}$. Then, it executes the forward and backward Phases. The forward (backward) Phase executes forward (backward) Iterations until some stopping criteria are met. Specifically, the forward Phase terminates if the maximum number of variables $maxVars$ has been selected, or until no more variable can be selected, while the backward Phase terminates if no more variables can be removed from $\mathbf {S}$.
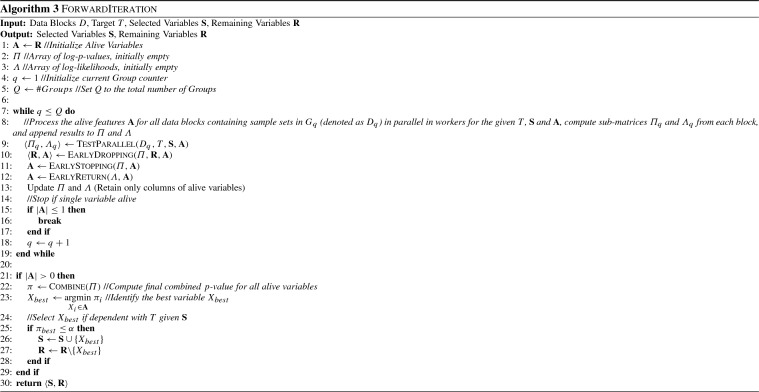

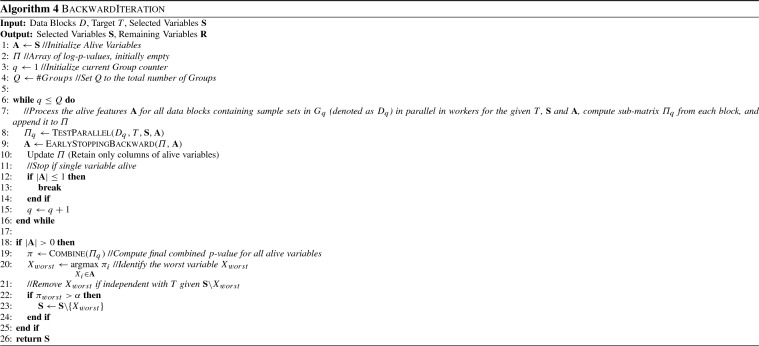


The forward and backward Iteration procedures are shown in Algorithms 3 and 4. $\textsc {ForwardIteration}$ takes as input the data blocks *D*, the target variable *T* as well as the current sets of remaining and selected variables, performs a forward Iteration and outputs the updated sets of selected and remaining variables. It uses the variable set $\mathbf {A}$ to keep track of all alive variables, i.e. variables that are candidates for selection. The arrays $\varPi$ and $\varLambda$ contain the local log *p*-values and log-likelihoods, containing $ns$ rows (one for each sample set) and $|\mathbf {A}|$ columns (one for each alive variable). The values of $\varPi$ and $\varLambda$ are initially empty, and are filled gradually after preprocessing each Group. We use $D_q$ to denote all data blocks which corresponds to Sample Sets contained in Group $G_q$. Similarly, accessing the values of $\varPi$ and $\varLambda$ corresponding to Group *q* and variables $\mathbf {X}$ is denoted as $\varPi _q$ and $\varLambda _q$.

In the main loop, the algorithm iteratively processes Groups in a synchronous fashion, until all Groups have been processed or no alive variable remains. The $\textsc {TestParallel}$ function takes as input the data blocks $D_q$ corresponding to the current Group $G_q$, and performs all necessary independence tests in parallel in workers. The results, denoted as $\varPi _q$ and $\varLambda _q$ are then appended to the $\varPi$ and $\varLambda$ matrices respectively. After processing a Group, the tests for Early Dropping, Early Stopping and Early Return are performed, using all local *p*-values computed up to Group *q*; details about the implementation of the EarlyDropping, EarlyStopping and EarlyReturn algorithms when data have only been partially processed are given in Sect. 4. The values of non-alive features are then removed from $\varPi$ and $\varLambda$ (see also Fig. 2f for an example). If only a single alive variable remains, processing stops. Note that, this is not checked in the while loop condition, in order to ensure that at least one Group has been processed if the input set of remaining variables contains a single variable. Finally, the best alive variable (if such a variable exists) is selected if it is conditionally dependent with *T* given the selected variables $\mathbf {S}$. Conditional dependence is determined by using the *p*-value resulting from combining all local *p*-values available in $\varPi$. $\textsc {BackwardIteration}$ is similar to ForwardIteration with the exception that (a) the remaining variables are not needed, and thus no dropping is performed, (b) no early return is performed, and (c) the tests are reversed, i.e. the worst variable is removed.

### Massively-parallel predictive modeling

The technique of combining locally computed *p*-values to global ones to massively parallelize computations, can be applied not only for feature selection, but also for predictive modeling. At the end of the feature selection process one could obtain an approximate predictive model with no additional overhead! We exploit this opportunity in the context of independence tests implemented by logistic regression. During the computation of local *p*-values $\pi _{i,k}$ a (logistic) model for *T* using all selected features $\mathbf {S}$ is produced from the samples in $S_i$. Such a model computes a set of coefficients $\beta _{i}$ that weighs each feature in the model to produce the probability that $T = 1$. We used the weighted univariate least squares (WLS) approach (Hedges and Vevea 1998), with equal weights for each model; equal weights were used as the sample size of each partition is (approximately) the same. The WLS method with equal weights combines the *N* local models to a global one $\hat{\beta }$ by just taking the average of the coefficient vectors of the model , i.e., $\hat{\beta } = \frac{1}{N}\sum _{i=1}^N \beta _i$. Thus, the only change to the algorithm is to cache each $\beta _{i}$ and average them in the master node this way. By default, PFBP uses the WLS method to construct a predictive model at each forward Iteration. Other multivariate methods for combining multiple models, which also consider the co-variance of the estimated coefficients are described in Becker and Wu (2007). Such methods could also be applied in our case without any significant computational overhead, but were not further considered in this work.

Using the previous technique, one could obtain a model at the end of each Iteration without extra computations and assess its predictive performance (e.g., accuracy) on a hold-out validation set. Constructing for instance the graph of the number of selected features versus achieved predictive performance on the hold-out set could visually assist data analysts (Konda et al. 2013) in determining how many features to include in the final selections; an example application on SNP data is given in the experimental section in Fig. 5. An automated criterion for selecting the best trade-off between the number of selected features and the achieved predictive performance could also be devised, although this is out of the scope of this paper, as multiple testing has to be taken into consideration.

## Implementation of the Early Dropping, Stopping and Return heuristics using bootstrap tests on local *p*-values

Recall that the algorithm processes Group Samples sequentially. After processing each Group and collecting the results, PFBP applies the Early Dropping, Early Stopping and Early Return heuristics, computed on the master node, to filter out variables and reduce subsequent computation. Thus, all three heuristics involve making early probabilistic decisions based on a subset of the samples examined so far. Naturally, if all samples have been processed, Early Dropping can be applied on the combined *p*-values without making probabilistic decisions.

Before proceeding with the details, we provide the notation used hereafter. Let $\varPi$ and $\varLambda$ be 2-dimensional arrays containing *K* local log *p*-values and log-likelihoods for all alive variables in $\mathbf {A}$ and for all Groups already processed. The matrices reside on the master node, and are updated each time a Group is processed. Let $\pi _{i,j}$ and $\lambda _{i,j}$ denote the *i*th value of the *j*th alive variable, denoted as $X_j$. Recall that those values have been computed locally on the Data Block containing samples from Sample Set $S_i$. For the sake of simplicity, we will use $\pi _j$ and $\lambda _j$ ($l_j$) to denote the combined *p*-value and sum of log-likelihoods (likelihood) respectively of variable $X_j$. The vectors $\pi$ and $\lambda$ will be used to refer to the combined *p*-values and sum of log-likelihoods for all alive variables respectively. Also, let $X_{best}$ be the variable that would have been selected if no more data blocks were evaluated, that is, the one with the currently lowest combined *p*-value, denoted as $\pi _{best}$.

### Bootstrap tests for early probabilistic decisions

In order to make early probabilistic decisions, we test: (a) $P(\pi _j \ge \alpha ) > P_{drop}$ for Early Dropping of $X_j$ (i.e., the probability of the *j*th feature deemed independent at significance level $\alpha$ at the end of the Iteration is larger than a threshold), (b) $P(\pi _{best} < \pi _j ) > P_{stop}$ for Early Stopping of $X_j$ (i.e., the probability of the current best feature having a smaller “better” *p*-value than feature *j* is larger than a threshold), and (c) $\forall X_j, (P(l_{best}/l_j \ge t) > P_{return})$ for Early Return of $X_{best}$ (the likelihood ratio $l_{best}/l_j$ indicate how close is the model with the currently best feature and the mode with feature $l_j$; if all ratios with all alive features are above a certain threshold with high probability, then the current best choice is close to optimal). The *t* is a tolerance parameter that determines how close the compared models should be. It takes values between 0 and 1; the closer it is to 1, the closer it is guaranteed that the current best model will be to all other ones in consideration in terms of likelihood. By taking the logarithm, (c) can be rewritten as $\forall X_j, P(\lambda _{best} - \lambda _j \ge lt)$, where $lt = \log (t)$.

We employed bootstrapping to test the above. A bootstrap-sample *b* of $\varPi$ ($\varLambda$), denoted as $\varPi ^b$ ($\varLambda ^b$), is created by sampling with replacement *Krows* from $\varPi$ ($\varLambda$). Then, for each such sample, the Fisher’s combined *p*-values (sum of log-likelihoods) are computed, by summing over all respective values for each alive variable; we refer to the vector of combined *p*-values (log-likelihoods) on bootstrap sample b as $\pi ^b$ ($\lambda ^b$), and the *i*th element is referred to as $\pi _i^b$ ($\lambda _i^b$). By performing the above *B* times, probabilities (a), (b) and (c) can be estimated as:

Early Dropping $$\begin{aligned} P(\pi _j \ge \alpha ) = \frac{{\textsc {I}}(\pi _j \ge \alpha ) + \sum _{b=1}^B {\textsc {I}}(\pi _j^b \ge \alpha )}{B + 1} \end{aligned}$$

Early Stopping $$\begin{aligned} P(\pi _j> \pi _{best}) = \frac{{\textsc {I}}(\pi _j> \pi _{best}) + \sum _{b=1}^B {\textsc {I}}(\pi _j^b > \pi _{best}^b)}{B + 1} \end{aligned}$$

Early Return $$\begin{aligned} P(\lambda _{best} - \lambda _j \ge lt) = \frac{{\textsc {I}}(\lambda _{best} - \lambda _j \ge lt) + \sum _{b=1}^B {\textsc {I}}(\lambda _{best}^b - \lambda ^b_j \ge lt)}{B + 1} \end{aligned}$$

where ${\textsc {I}}$ is the indicator function, which evaluates to 1 if the inequality holds and to 0 otherwise. For all of the above, the condition is also computed on the original sample, and the result is divided by the number of bootstrap iterations *B* plus 1. Note that, for Early Return the above value is computed for all features $X_j$.

Algorithms 5, 6 and 7 show the procedures in more detail. For all heuristics, a vector named *cnts* is used to keep track of how often the inequality is satisfied for each variable. To avoid cluttering, the indicator function ${\textsc {I}}$ performs the check for multiple variables and returns a vector of values in each case, containing one value for each variable. The function ${\textsc {BootstrapSample}}$ creates a bootstrap sample as described above, function ${\textsc {Combine}}$ uses Fisher’s combined probability test to compute a combined *p*-value, and ${\textsc {SumRows}}$ sums over all rows of the log-likelihoods contained in $\varLambda$, returning a single value for each alive variable.
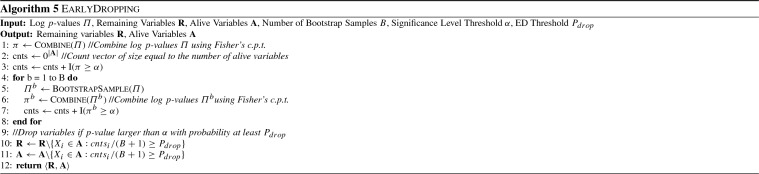

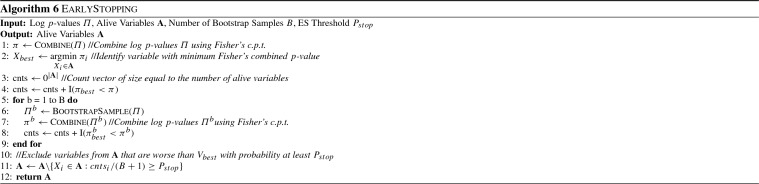

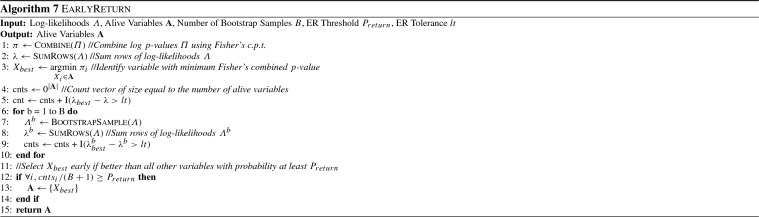


### Implementation details of bootstrap testing

We recommend using the same sequence of bootstrap indices for each variable, and for each bootstrap test. The main reasons are to (a) simplify implementation, (b) avoid mistakes and (c) ensure results do not change across different executions of the algorithms. This can be done by initializing the random number generator with the same seed. Next, note that ED, ES and ER do not necessarily have to be performed separately, but can be performed simultaneously (i.e,. using the same bootstrap samplings). This allows the re-usage of the sampled indices for all tests and variables, saving some computational time. Another important observation for ED and ES is that the actual combined *p*-values are not required. It suffices to compare statistics instead, which are inversely related to *p*-values: larger statistics correspond to lower *p*-values. For the ED test, the statistic has to be compared to the statistic corresponding to the significance level $\alpha$, which can be computed using the inverse $\chi ^2$ cumulative distribution. This is crucial to speed-up the procedure, as computing log *p*-values is computationally expensive. Finally, note that it is not always necessary to perform all bootstrap iterations to decide if the probability is below the threshold. This can be done by keeping track of an upper bound of the estimated probabilities, and to stop the bootstrap procedure if that bound is below the threshold, further reducing the computational cost. For example, let $P_{drop} = 0.99$ and $B = 999$. Then, in order to drop a variable $X_i$, the number of times $cnts_i$ where the *p*-value of $X_i$ exceeds $\alpha$ has to be at least 990. If after *K* iterations $(B - K) + cnts_i$ is less than 990, one can determine that $X_i$ will not be dropped; even if in all remaining bootstrap iterations its *p*-value is larger than $\alpha$, $cnts_i + B - K$ will always be less than 990, and thus the probability $P(\pi _{i} \ge \alpha )$ will be less than the threshold $P_{drop} = 0.99$.

Finally, we note that, in order to minimize the probability of wrong decisions, large values for the ED, ES and ER thresholds should be used. We found that values of 0.99 for $P_{drop}$ and $P_{stop}$, and values of $P_{return} = 0.95$ and $tol = 0.9$ work well in practice. Furthermore, the number of bootstraps *B* should be as large as possible, with a minimum recommended value of 500. By default, PFBP uses the above values.

## Tuning the data partitioning parameters of the algorithm

The main parameters for the data partitioning to determine are (a) the sample size *s* of each Data Block, (b) the number of features *f* in each Data Block, and (c) the number of Sample Subsets *C* in each Group; the latter determines how many new *p*-values per feature are computed in each Group. Notice that *s determines the horizontal partitioning of the data matrix and f the vertical partitioning of data matrix*. In this section, we provide detailed guidelines to determine those parameters, and show how those values were set for the special case of PFBP using conditional independence tests based on binary logistic regression. *Selecting the data partitioning parameters needs to consider both statistical phenomena, as well as the hardware architecture*. A trade-off exists between accuracy of statistical estimation of *p*-values and the bootstrap tests and the induced parallelism.

### Determining the required sample size *s* for conditional independence tests

For optimal computational performance, the number of Sample Sets should be as large as possible to increase parallelism, and each Sample Set should contain as few samples as possible to reduce the computational cost for performing the local conditional independence tests. On the other hand, there should be enough samples per Sample Set so that the local tests have enough statistical power.

Various rules of thumb have appeared in the literature to choose a sufficient number of samples for linear, logistic and Cox regression (Peduzzi et al. 1996; Harrell 2001; Vittinghoff and McCulloch 2007). We focus on the case of binary logistic regression hereafter. For binary logistic regression, it is recommended to use at least $s = c/\min (p_0,p_1) \cdot df$ samples, where $p_0$ and $p_1$ are the proportion of negative and positive classes in *T* respectively, *df* is the number of degrees of freedom in the model (that is, the total number of parameters, including the intercept term) and *c* is usually recommended to be between 5 and 20, with larger values leading to more accurate results. This rule is based on the events per variable (EPV) (Peduzzi et al. 1996), and will referred to as the EPV rule hereafter.

Rules like the above can be used to determine the number of samples *s* in each Sample Set, by setting the minimum number of samples in each Data Block in a way that the locally computed *p*-values are valid for the type of test employed *in the worst case*. The worst case scenario occurs if the maximum number of features $maxVars$ have been selected. If all features are continuous, then the maximum number of parameters of a model is $df = maxVars + 1$. This can easily be adapted for the case of categorical features, by considering the $maxVars$ variables with the most categories, and setting *df* appropriately. By considering the worst case scenario, the required number of samples can be computed by plugging the values of *df*, *c*, $p_0$ and $p_1$ into the EPV rule. We found out that, although the EPV rule works reasonably well, it tends to overestimate the number of samples required for skewed class distributions. As a result, it may unnecessarily slow down PFBP in such cases. Ideally for a given value of *c* the results should be equally accurate irrespective of the class distribution and the number of model parameters.

To overcome the drawbacks of the EPV rule, we propose another rule, called the STD rule, which is computed as $s = df \cdot c/\sqrt{p_0 \cdot p_1}$. For balanced class distributions the result is identical to the EPV rule, while for skewed distributions the value is always smaller. We found that a value of $c = 10$ works sufficiently well, and recommend to always set *c* to a minimum of 10; higher values could lead to more accurate results, but will also increase computation time. Again, the number of samples per Sample Set is determined as described above. A comparison of both rules is given in “Appendix A”. We show that the STD rule behaves better across different values of *df* and class distributions of the outcome than the EPV rule.

### Setting the number of sample sets *C* per group

We now discuss the determination of the *C* value, the number of Sample Sets in each Group. The value of *C* determines how many Sample Sets are processed in parallel and thus, how many additional local *p*-values for each feature are added to matrix $\varPi$ at the end of processing each Group. In other words, before invoking the next round of bootstrap tests that decide on Early Dropping, Stopping or Return, *C* additional *p*-values will be available to these tests. We recommend a minimal value for *C* to be at least 15, otherwise the first round of bootstrap tests becomes unreliable. The value of *C* determines how often the workers stop and await the master to perform the bootstrap tests, which should not be too often. In our experiments we have set $C = 30$, without extensive tuning. In addition, whenever there is no progress made by any of the heuristics (when the “easy-to-determine” features have already been stopped or dropped), the value of *C* is doubled dynamically for that Iteration. This trick avoids stopping too often without any progress made. *C* is then reset in the next iteration.

### Determining the number of features per data block

At this point, we assume we have chosen the sample size *s* of each data block $D_{i,j}$. We also assume we have decided upon the value of *C*, i.e., the number of Sample Sets in each Group. In other words, we have selected the horizontal partitioning of the data at two levels: first, the partitioning of samples to Sample Sets and then to samples that belong to the same Group. Next, we need to decide the vertical partitioning to $nf$ equal-size Feature Sets. The number of blocks per group will then become $C \times nf$. In a system with *M* available workers that can process the blocks in parallel, it makes sense to determine $nf$ so that $M \approx C \times nf$. Specifically, we set $nf = \lfloor M / C \rfloor$. In the extreme case where a data block does not fit in the main memory of a machine, $nf$ has be to increased and the data to be physically partitioned to different machines.

## Practical considerations and implementation details

In this section, we discuss several important details for an efficient and accurate implementation of PFBP. The main focus is on PFBP using conditional independence tests based on binary logistic regression, which is the test used in the experiments, although most details regard the general case or can be adapted to other conditional independence tests.

### Accurate combination of local *p*-values using Fisher’s method

In order to apply Fisher’s combined probability test, *the data distributions of each data block should be the same for the test to be valid*. There should be no systematic bias on the data or the combining process may exacerbate this bias [see Tsamardinos and Mariglis (2009)]. Such bias may occur if blocks contain data from the same departments, stores, or branches, or in consecutive time moments and there is time-drift on the data distribution. This problem is easily avoided if before the analysis the partitioning of samples to blocks is done randomly, as done by PFBP.

Another important detail to observe in practice, is to *directly compute the logarithm of thep*-*values for each conditional independence test* instead of first computing the *p*-value and then taking the logarithm. As *p*-values tend to get smaller with larger sample sizes (in case the null hypothesis does not hold), they quickly reach the machine epsilon, and will be rounded to zero. If this happens, then sorting and selecting features according to *p*-values breaks down and PFBP will select an arbitrary feature. This behavior is further magnified in case of combined *p*-values, as a single zero local *p*-value leads to a zero combined *p*-value no matter the values of the remaining *p*-values. The R language provides the option to directly compute the logarithm of the *p*-value (using the option $log.p=T$). Next, we give pointers to our implementation, since there was no other implementation available for Spark. For the $\chi ^2$ distribution, the *p*-value can be computed as $\frac{\varGamma (k/2,x/2)}{\varGamma (k/2)}$, where *x* is the test statistic, *k* the degrees of freedom, $\varGamma (\cdot ,\cdot )$ the incomplete gamma function and $\varGamma (\cdot )$ the gamma function. Formulas for computing the incomplete gamma function can be found in Chaudhry and Zubair (2001) (equation 2.27 for $k/2 = n + 1/2, n = 0, 1, 2, \dots$ and corollary 2.1 for positive integer values of *k* / 2). By careful computation of the terms of the sums, the logarithm of the *p*-value can be computed with very high accuracy (even $10^{-1000000}$).

### Implementation of the conditional independence test using logistic regression for binary targets

The conditional independence test is the basic building block of PFBP, and thus using a fast and robust implementation is essential. Next, we briefly review optimization algorithms used for maximum likelihood estimation, mainly focusing on binary logistic regression, and in the context of feature selection using likelihood-ratio tests.

A comprehensive introduction and comparison of algorithms for fitting (i.e., finding the $\beta$ that maximizes the likelihood) binary logistic regression models is provided in Minka (2003). Three important classes of optimization algorithms are Newton’s method, conjugate gradient descent and quasi-Newton methods. Out of those, Newton’s method is the most accurate and typically converges to the optimal solution in a few tens of iterations. The main drawback is that each such iteration is slow, requiring $O(n \cdot d^2)$ computations, where *n* is the sample size and *d* the number of features. Conjugate gradient descent and quasi-Newton methods on the other hand require $O(n \cdot d)$ and $O(n \cdot d + d^2)$ time per iteration, but may take much longer to converge. Unfortunately, there are cases were those methods fail to converge to an optimal solution even after hundreds of iterations. This not only affects the accuracy of feature selection, but also leads to unpredictable running times. Most statistical packages include one or multiple implementations of logistic regression. Such implementations typically use algorithms that can handle thousands of predictors, with quasi-Newton methods being a popular choice. For feature selection however, one is typically interested to select a few tens or hundreds of variables. In anecdotal experiments, we found that for this case Newton’s method is usually faster and more accurate, especially with fewer than 100–200 variables. Because of that, and because of the issues mentioned above, we used a fine-tuned, custom implementation of Newton’s method.

There are some additional, important details. First of all, there are cases where the Hessian is not invertible.^11^ If this the case, we switch to conjugate gradient descent using the fixed Hessian as a search direction for that iteration, as described in Minka (2003). Finally, as a last resort, in case the fixed Hessian is not invertible we switch to simple gradient descent. Next, for all optimization methods there are cases in which the computed step-size has to be adjusted to avoid divergence, whether it is due to violations of assumptions or numerical issues. One way to do this is to use inexact line-search methods, such as backtracking-Armijo line search (Armijo 1966), which was used in our implementation.

### Score tests for the univariate case

In the first step of forward selection where no variable has been selected, one can use a score test (also known as Lagrange multiplier test) instead of a likelihood-ratio test to quickly compute the *p*-value without having to actually fit logistic regression models. The statistic of the Score test equals (Hosmer et al. 2013)

$$\begin{aligned} \text {Statistic} \equiv \frac{\sum _{j=1}^{n} X_j (T_j - \bar{T})}{\sqrt{{\bar{T}(1 - \bar{T})\sum _{j=1}^{n} (X_j - \bar{X})^2}}} \end{aligned}$$

where *n* is the number of samples, *T* is the binary outcome variable (using a 0/1 encoding), and *X* is the variable tested for independence. Note that, such tests can also be derived for models other than binary logistic regression, but it is out of the scope of the paper. The score test is asymptotically equivalent to the likelihood ratio test, and in anecdotal experiments we found that a few hundred samples are sufficient to get basically identical results, justifying its use in Big Data settings. Using this in place of the likelihood ratio test reduces the time of the univariate step significantly and is important for an efficient implementation, as the first step is usually the most computationally demanding one in the PFBP algorithm, as a large portion of the variables will be dropped by the Early Dropping heuristic.

## Optimality of PFBP on distributions faithful to Bayesian networks and maximal ancestral graphs

Assuming an oracle of conditional independence, it can be shown that the standard Forward–Backward Selection algorithm is able to identify the optimal set of features for distributions faithful to Bayesian networks or maximal ancestral graphs (Margaritis and Thrun 2000; Tsamardinos et al. 2003b; Borboudakis and Tsamardinos 2017). Unfortunately, the Early Dropping (ED) heuristic (without further adjustments) may compromise the optimality of the method. ED may remove features that are necessary for optimal prediction of *T*. Intuitively, these features provide no predictive information for *T* given $\mathbf {S}$ (are conditionally independent) but become conditionally *dependent* given a superset of $\mathbf {S}$, i.e., after more features are selected. This problem can be overcome by using multiple Runs of the Forward–Backward Phases. Recall that, each Run reinitializes the remaining variables with $\mathbf {R} = \mathbf {F} {\setminus } \mathbf {S}$. Thus, each subsequent Run provides each feature with another opportunity to be selected, even if it was Dropped in a previous one. The heuristic has a graphical interpretation in the context of probabilistic graphical models such as Bayesian networks and maximal ancestral graphs (Pearl 2000; Spirtes et al. 2000; Richardson and Spirtes 2002) inspired by modeling causal relations. A rigorous treatment of the Early Dropping heuristic and theorems regarding its optimality for distributions faithful to Bayesian networks and maximal ancestral graphs is provided in Borboudakis and Tsamardinos (2017); for the paper to be self-sustained, we provide the main theorems along with proofs next.

We assume that PFBP has access to an *independence oracle* that determines whether a given conditional dependence or independence holds. Furthermore, we assume that the Markov and faithfulness conditions hold, which allow us to use the terms d-separated/m-separated and independent (dependent) interchangeably. We will use the the *weak union* axiom, one of the *semi-graphoid* axioms (Pearl 2000) about conditional independence statements, which are general axioms holding in all probability distributions. The weak union axiom states that $\mathbf {X} {\perp } \mathbf {Y}\cup \mathbf {W} \ | \ \mathbf {Z} \Rightarrow \mathbf {X} {\perp } \mathbf {Y} \ | \ \mathbf {Z}\cup \mathbf {W}$ holds for any such sets of variables.

### Theorem 1

If the distribution can be faithfully represented by a Bayesian network, then PFBP with two Runs identifies the Markov blanket of the target *T*.

### Proof

In the first run of PFBP, all variables that are adjacent to *T* (that is, its parents and children) will be selected, as none of them can be d-separated from *T* by any set of variables. In the next run, all variables connected through a collider path of length 2 (that is, the spouses of *T*) will become d-connected with *T*, since the algorithm conditions on all selected variables (including its children), and thus spouses will be selected as at least a *d*-connecting path is open: the path that goes through the selected child. The resulting set of variables includes the Markov blanket of *T*, but may also include additional variables. Next we show that all additional variables will be removed by the backward selection phase. Let MB(*T*) be the Markov blanket of *T* and $\mathbf {S_{ind}} = \mathbf {S} {\setminus }$MB(*T*) be all selected variables not in the Markov blanket of *T*. By definition, $T {\perp } \mathbf {X} \ | \ MB(T)$ holds for any set of variables $\mathbf {X}$ not in MB(*T*), and thus also for variables $\mathbf {S_{ind}}$. By applying the weak union graphoid axiom, one can infer that $\forall S_i \in \mathbf {S_{ind}}, T {\perp } S_i \ | \ MB(T) \cup \mathbf {S_{ind}} {\setminus } S_i$ holds, and thus some variable $S_j$ will be removed in the first iteration. Using the same reasoning and the definition of a Markov blanket, it can be shown that all variables in $\mathbf {S_{ind}}$ will be removed from MB(*T*) at some iteration. To conclude, it suffices to use the fact that variables in MB(*T*) will not be removed by the backward selection, as they are not conditionally independent of *T* given the remaining variables in MB(*T*). $\square$

### Theorem 2

If the distribution can be faithfully represented by a directed maximal ancestral graph, then PFBP with no limit on the number of Runs identifies the Markov blanket of the target *T*.

### Proof

In the first run of PFBP, all variables that are adjacent to *T* (that is, its parents, children and variables connected with *T* by a bi-directed edge) will be selected, as none of them can be m-separated from *T* by any set of variables. After each run additional variables may become admissible for selection. Specifically, after *k* runs all variables that are connected with *T* by a collider path of length *k* will become m-connected with *T*, and thus will be selected; we prove this next. Assume that after *k* runs all variables connected with *T* by a collider path of length at most $k-1$ have been selected. By conditioning on all selected variables, all variables with edges into some selected variable connected with *T* by a collider path will become m-connected with *T*. This is true because conditioning on a variable *Y* in a collider $\langle X, Y, Z \rangle$ m-connects *X* and *Z*. By applying this on each variable on some collider path, it is easy to see that its end-points become m-connected. Finally, after applying the backward selection phase, all variables that are not in the Markov blanket of *T* will be removed; the proof is identical to the one used in the proof of Theorem 1. $\square$

## Related work

In this section we provide an overview of related parallel feature selection methods, focusing on methods for MapReduce-based systems (such as Spark), as well as causal-based methods, and compare them to PFBP. An overview of feature selection methods can be found in Guyon and Elisseeff (2003) and Li et al. (2017).

### Parallel univariate feature selection and parallel Forward–Backward selection

UFS and FBS rank features according to *p*-values of independence tests. UFS computes the *p*-values of the unconditional test between a feature and the target, while FBS performs conditional tests given the already selected features. UFS statically ranks features, while FBS updates the ranking with every newly selected feature. The algorithms stop when the maximum number of features has been selected, or the *p*-values are below some significance threshold. *Both UFS and FBS can be parallelized at the level of the underlying statistical test employed*. Specifically, the Spark machine learning library MLlib (Meng et al. 2016) offers parallel implementations of Pearson and Spearman correlation coefficients for continuous data, and the chi-square test of independence for discrete data. For conditional independence, tests can be constructed using the likelihood ratio technique by fitting statistical models. MLlib offers parallelized binomial, multinomial, and linear regression models to this end. We employed these parallelized statistical tests to implement UFS and FBS in the experimental section.

The main advantages of PFBP over UFS and FBS are that (a) PFBP does not require specialized distributed implementations of independence tests, as it only relies on local computations and thus can use existing implementations. Local fitting and combining is also much faster than fitting full models over all samples, and (b) PFBP employs the Early Dropping, Early Stopping and Early Return heuristics, allowing it to scale both with number of features and samples. Perhaps, most importantly (c) UFS will not necessarily identify the Markov Blanket of *T* even in faithful distributions; the solution by UFS will have false positives (e.g., redundant features) as well as false negatives (missed Markov Blanket features).

### Single feature optimization

The Single Feature Optimization algorithm (SFO) (Singh et al. 2009) is a Map-Reduce-based extension of the standard forward selection algorithm using binary logistic regression. In essence, SFO employs an efficiency trick to approximate the parallel computation of the criterion to select the next best feature, when the binary logistic regression test is employed. Thus, the algorithm is specific to classification tasks and cannot be tivially generalized to other types of classifiers in place of the logistic regression.

In more detail, SFO (a) employs a heuristic that ranks the features at each step without the need to fit a full logistic regression model (that is, one over all samples) for all variables, and (b) uses a parallelization scheme to perform parallel computation over samples and features.

We proceed by describing the ranking heuristic used by SFO. Let $\mathbf {S}$ be the selected features up to some point and $\mathbf {R} = \mathbf {F} {\setminus } \mathbf {S}$ be all candidate variables for selection, and assume that a full logistic regression model *M* for *T* using $\mathbf {S}$ is available. SFO creates an approximate model for each variable $R_i \in \mathbf {R}$ by fixing the coefficients of $\mathbf {S}$ using their coefficients in *M*, and only optimizing the coefficient of $R_i$. This problem is much simpler than fitting full models for each remaining variable, significantly reducing running time. Then, the best variable $R^*$ is chosen based on those approximate models (using some performance measure such as the log-likelihood), and a full logistic regression model $M^*$ with $\mathbf {S} \cup R^*$ is created. Thus, at each iteration only a single, full logistic regression model needs to be created. By default, SFO uses a maximum number of variables to select as a stopping criterion. Alternatively, to decide whether $R^*$ should be selected a likelihood-ratio test could be used, in which case the test is performed on *M* and $M^*$, and $R^*$ is selected if the *p*-value is below a threshold $\alpha$; we used this in our implementation of SFO in the experiments. The parallelization over samples is performed in the map phase, in which one value $p_j$ is computed for each sample *j*, which equals

$$\begin{aligned} p_j = \frac{e^{\beta \cdot \mathbf {S}_j}}{1 + e^{\beta \cdot \mathbf {S}_j}} \end{aligned}$$

where $\beta$ are the coefficients of $\mathbf {S}$ in *M* and $\mathbf {S}_j$ are the values of $\mathbf {S}$ in the *j*th sample. The values of $p_j$, the values of the outcome *T* and all of candidate variables $\mathbf {R}$ are then sent to workers to be processed in the reduce phase. Note that, *this incurs a high communication cost, as essentially the whole dataset has to be exchanged among workers over the network*. Finally, in the reduce phase, all workers fit in parallel over all variables $\mathbf {R}$ the approximate logistic regression models.

Although SFO significantly improves over the standard forward selection algorithm in terms of running time, it has three main drawbacks compared to PFBP: (a) it has a high communication cost, in contrast to PFBP which only requires minimal information to be communicated, (b) to select a variable all non-selected variables have to be considered, while PFBP employs the Early Dropping heuristic that significantly reduces the number of remaining variables, and (c) SFO always uses all samples, while PFBP uses Early Stopping and Early Return allowing it to scale sub-linearly with number of samples. Finally, (d) SFO does not provide any theoretical guarantees of correctness.

### Information theoretic feature selection for Big Data

Information theoretic feature selection (ITFS) (Brown et al. 2012) methods have been extended to Big Data settings (Ramrez-Gallego et al. 2017) and implemented for Spark.^12^ They are applicable only for discrete features and outcomes; non-discrete data would have to be discretized. ITFS relies on *estimations* of the mutual information and the conditional mutual information (CMI), and many variations have appeared in the literature (Brown et al. 2012); we provide a brief description next. The criterion *J* of several ITFS methods^13^ for evaluating feature $X_k$ can be expressed as

$$\begin{aligned} J(X_k) = I(T;X_k) - \beta \sum _{X_j \in \mathbf {S}} I(X_j;X_k) + \gamma \sum _{X_j \in \mathbf {S}} I(X_j;X_k|T) \end{aligned}$$

where $\beta$ and $\gamma$ are parameters taking values in [0, 1], and *I* denotes the mutual or conditional mutual information. The intuition for $J(X_k)$ above, is that *J* increases with the information $X_k$ directly provides for the target *T* (the first term), decreases with the information the other selected features already provide for $X_k$ (second group of terms), and increases when $X_k$ interacts with the selected features, i.e., one provides information for the other features conditioned on *T* (third group of terms). *ITFS methods also perform a greedy type of forward selection, adding a feature at a time*, albeit with a different selection criterion than PFBP. The next best feature is chosen as the one maximizing *J* with respect to the current set of selected variables $\mathbf {S}$.

Specifically, for the implementations examined in this paper, ITFS estimate CMIs assuming a multinomial model of the joint between (discrete) features. In this case, the estimated MI $I(X;Y) = \frac{1}{2}G^2(X;Y)$, where $G^2(X;Y)$ is the test statistic of a $G^2$ likelihood ratio test. In other words, the estimations correspond to using a $G^2$ likelihood ratio test of conditional independence in statistical-based FS algorithm (Agresti 2002). In contrast, the current implementation of PFBP uses a logistic regression test that imposes a linear dependence of the probabilities of the joint to the values of the features.

The main advantage of the specific implementation of ITFS over PFBP’s is that estimating the (conditional) mutual informations for discrete data under the multinomial assumption, does not require fitting any model; it only requires counting and constructing the contingency tables of the joint. This can be done in one pass of the data and can thus trivially exploit the sparsity of the data. In Big Data settings it can be easily parallelized. However, ITFS methods do not have the same theoretical properties of PFBP, which can be shown to be optimal for distributions that can be faithfully represented by Bayesian networks and maximal ancestral graphs. This stems from the fact that PFBP solves an inherently harder problem, as it conditions on all selected features (creates a model with all selected features) at each iteration in order to select the next feature, while ITFS only conditions on one feature at a time. Furthermore, ITFS methods are not as general as PFBP, which can be applied to various data types as long as an appropriate conditional independence test is available. For example, it is not clear if and how ITFS can be applied to time-to-event outcome variables or time-course data. Instead, appropriate statistical tests for these cases are available and put in use within statistical-based methods similar to PFBP (Lagani et al. 2017). Last but not least, as currently implemented, ITFS variants are only applicable to discrete data. Thus, in case of continuous variables, a discretization method has to be applied before feature selection, possibly losing information (Kerber 1992; Dougherty et al. 1995). This not only increases computational time but also may require extra tuning to find a good discretization of features.

### Lasso, Orthogonal Matching Pursuit, Least Angle Regression, and Forward Stagewise Regression

Given that the problem of FS in general is NP-Hard, it is natural that any algorithm that scales to high-dimensional data employs some approximations or heuristics that are sound only in a restricted class of distributions. A large class of algorithms, namely the *Lasso* (Tibshirani 1996), *Least Angle Regression* (Efron et al. 2004), *Forward Stagewise Regression* (Efron et al. 2004) *and Orthogonal Matching Pursuit* (Pati et al. 1993; Davis et al. 1994) are all greedy-like versions of the basic Forward–Backward FS. A detailed comparison between Lasso, Least Angle Regression and Forward Stagewise Regression can be found in Efron et al. (2004), a comparison between Least Angle Regression and Orthogonal Matching Pursuit is given in Hameed (2012), while a comparison between Orthogonal Matching Pursuit and Forward Selection (called Orthogonal Least Squares) can be found in Blumensath and Davies (2007). We proceed with a brief high-level comparison of the above with the Forward Selection algorithm.

All of the above algorithms select the next feature using some selection criterion and are equipped with a stopping criterion. On an intuitive level, they select as the next feature to include *the feature that provides the most information (highest correlation) for the errors (residuals) of the current model. In contrast, Forward Selection and PFBP select the feature that provides the most additional information for the target (given all other selected features)*. In Lasso, the Forward–Backward phases are interleaved. When a feature is selected for inclusion forward selection, PFBP, and Orthogonal Matching Pursuit construct a new unrestricted model that includes the newly selected feature. Lasso, Least Angle Regression and Forward Stagewise Regression also create a new model, but constrain the coefficients of the newly selected features. Lasso has a special stopping criterion based on the L1-norm of the coefficients of the current selections. *Given this synthetic view and connections between the algorithms, we would like to note that the ideas, techniques, and heuristics presented, could be translated for use with several other prominent FS algorithms*.

We now focus in more detail on Lasso (Tibshirani 1996), which is perhaps one of the most widely used FS algorithm. The feature selection problem is expressed as a global optimization problem using an $L_1$ penalty on the feature coefficients. Let $D(\theta )$ be the deviance of a (generalized linear) model using *n* parameters $\theta$. The optimization problem Lasso solves can be expressed as

$$\begin{aligned} \min _{\theta \in \mathbb {R}^n} D(\theta ) + \lambda \left||\theta \right||_{1} \end{aligned}$$

where $\left||\theta \right||_{1}$ is the $L_1$ norm and $\lambda \ge 0$ is a regularization parameter. The solutions Lasso returns are sparse, meaning that most coefficients are set to zero, thus implicitly performing feature selection. The regularization parameter $\lambda$ controls the number of non-zero coefficients in the solution, with larger values leading to sparser solutions. This problem formulation is a convex approximation of the more general best subset selection (BSS) problem (Miller 2002), defined as follows to match the Lasso optimization formulation

$$\begin{aligned} \min _{\theta \in \mathbb {R}^n} D(\theta ) + \lambda \left||\theta \right||_{0} \end{aligned}$$

where $\left||\theta \right||_{0}$ is the 0-norm (i.e., the total number of variables with non-zero coefficients). The BSS problem has been shown to be NP-hard (Welch 1982), and thus most approaches, such as Lasso and forward selection, rely on some type of approximation to solve it.^14^ A sufficient condition for optimality of PFBP and FBS to solving the BSS (equivalent to finding the Markov Blanket) is that distributions can be faithfully represented by Bayesian networks or maximal ancestral graphs (see Sect. 7). Conditions for optimal feature selection with Lasso are given in Meinshausen and Bühlmann (2006). *While Lasso is defined as a global optimization problem, it has been proven that many problems can be solved with a greedy Forward–Backward procedure* [e.g., for linear regression (Efron et al. 2004)].

Comparing Lasso with algorithms related to PFBP, we note that in extensive simulations it has been shown that causally-inspired feature selection methods are competitive in terms of predictive performance with Lasso on classification and survival analysis tasks on many real datasets (Aliferis et al. 2010; Lagani and Tsamardinos 2010; Lagani et al. 2013, 2017). Furthermore, the non-parallel version of PFBP (called Forward–Backward Selection with Early Dropping) as well as the standard Forward–Backward Selection algorithm have been shown to perform as well as Lasso when restricted to select the same number of variables (Borboudakis and Tsamardinos 2017). Finally, we note that in contrast to forward selection using conditional independence tests, Lasso is not easily extensible for different tasks, and requires specialized algorithms for different data types (Meier et al. 2008; Schelldorfer et al. 2011; Ivanoff et al. 2016), whose objective function may be non-convex (Schelldorfer et al. 2011) or computationally demanding (Fan et al. 2010). For example, for time-course data, the Lasso problem is not convex and does not scale up, while causal-based FS methods do (Tsagris et al. 2018).

Coming back to Big Data settings, the Lasso has been parallelized for single machines and shared-memory clusters (Bradley et al. 2011; Zhimin et al. 2013; Li et al. 2016). These approaches only parallelize over features and not samples (i.e. consider vertical partitioning). Naturally, ideas and techniques presented in those works could be adapted or extended for Spark or related systems. An implementation of Lasso linear regression is provided in the Spark MLlib library (Meng et al. 2016). A disadvantage of that implementation is that it requires extensive tuning of its hyper-parameters (like the regularization parameter $\lambda$ and several parameters of the optimization procedure), rendering it impractical as typically many different hyper-parameter combinations have to be tried to obtain the optimal settings. Unfortunately, we were not able to find any Spark implementation of Lasso for logistic regression, or any work dealing with the problem of efficient parallelization of Lasso on Spark.

### Other approaches

In addition to forward-selection based, information theoretic and Lasso based methods, there also exist other parallel feature selection methods which can’t directly be categorized into one of the above but are worth mentioning. However, none of those methods has actually been applied to Big Data, which may contain millions of samples and/or features. Furthermore, all of the methods perform virtual partitioning of the data, and thus only parallelize over features and not over samples, in contrast to PFPB which parallelizes over both.

Bolón-Canedo et al. (2017) introduced a method which uses vertical partitioning to distribute computations across workers. The method focuses mainly on DNA microarray data, whose number of features is much larger than the number of samples, although it is not limited to those cases. It can be applied using any feature selection method that ranks features, like the ITFS methods described previously, or even forward selection type algorithms using as a ranking the order of selection of variables. The main idea is to rank variables on each worker independently and to combine the partial rankings into a complete one. In order to be able to combine the rankings, each variable may be distributed to multiple workers in order to have some kind of overlap between the partial rankings, allowing them to be merged into one. Although the method is quite general, as it can be used in combination with any method that produces a ranking of the features, its theoretical properties are not explored in the paper, making it hard to compare theoretically against other methods.

Zhou et al. (2014) introduced a general, parallel feature selection method for classification tasks that is based on group testing theory. The idea is to select a collection of tests (i.e., a subset of the input variables), with each variable being present with some probability *p*. This implicitly creates multiple datasets, one for each test. Those datasets can be processed independently in parallel, producing a score using some scoring function for each dataset corresponding to how predictive the respective variables are of the outcome of interest. Then, variables are ranked based on the scores attained on the datasets they participated in. The authors show that, for specific values of *p*, if the number of tests is large enough and if the scoring function satisfies some properties (i.e., it is what the authors call *C*-separable), the proposed algorithm is able to select the best features with high probability. However, it is not clear how those results are related to the theoretical properties of other feature selection algorithm (such as PFBP or Lasso), nor how they can be used in practice, especially given that the authors do not propose any way to tune the hyper-parameters of their method.

Wang et al. (2016) proposed a method that also uses vertical partitioning for parallelization. It is method that can be used with any penalized regression method, both for feature selection and for model fitting. Given a dataset, it is partitioned vertically into multiple datasets, and a decorrelation step is performed on each new dataset that tries to minimize the dependencies between all datasets. Then, each dataset can be analyzed independently across multiple workers, and the results are combined into a final one on the master machine. It is shown that the convergence rate of the proposed method is nearly minimax optimal under weakly sparse assumptions on the model parameters. Furthermore, it is shown that the algorithms retains those properties irrespective of the number of partitions. In experiments, the method is shown to perform similarly to lasso while exhibiting lower running time. In contrast to PFBP, the method is specialized only to specific cases (penalized regression methods with continuous predictors), and thus is not easily extensible to other cases.

### Connections to Markov blanket based feature selection

Several algorithms have appeared in the literature that apply tests of conditional independence to select features. The theoretical properties of these algorithms often rely on the theory of Bayesian networks and the Markov blanket. The GS (Margaritis and Thrun 2000; Margaritis 2009) and the IAMB (Tsamardinos et al. 2003b) algorithms, were some of the first to present the Forward–Backward selection algorithm in the context of Bayesian networks and the Markov blanket and prove correctness for faithful distributions. These algorithms perform tests of independence conditioning each time on the full set $\mathbf {S}$ of selected features and can guarantee to identify the Markov blanket for faithful distributions asymptotically. However, the larger the conditioning set, the more samples are required to obtain valid results. Thus, these algorithms are not well-suited for problems with large Markov blankets relative to the available sample size.

Another class of such algorithms includes HITON-PC (Aliferis et al. 2003), MMPC (Tsamardinos et al. 2003a), and more recently SES (Lagani et al. 2017) for multiple solutions. The main difference in this class of algorithms is that they condition on *subsets of the selected features*$\mathbf {S}$, not the full set. They do not guarantee to identify the full Markov blanket, but only a superset of the parents and children of *T*. Recent extensive experiments have concluded that they perform well in practice (Aliferis et al. 2010). These algorithms remove from consideration any features that become independent of *T* conditioned on *some* subset of the selected features $\mathbf {S}$. This is similar to the Early Dropping heuristic and renders the algorithms quite computationally efficient and scalable to high-dimensional settings.

PFBP combines the advantages of these two classes of algorithms: those that condition on subsets, drop features from consideration and achieve scalability, and those that condition on the full set of selected features and guarantee identification of the full Markov blanket.

## Experimental evaluation

We performed three sets of experiments to evaluate PFBP.

1. We investigate the scalability of PFBP in terms of variable size, sample size and number of workers on synthetic datasets, simulated from Bayesian networks.
2. We compare PFBP to three competing forward-selection based feature selection algorithms.
3. We compare against three algorithms from the family of information theoretic feature selection methods (Brown et al. 2012), implemented for Big Data (Ramrez-Gallego et al. 2017).
4. We performed a proof-of-concept experiment of PFBP on dense synthetic Single Nucleotide Polymorphism (SNP) data. These are important types of very high-dimensional data that arise in biology and for which feature selection algorithms that can scale up are needed.

We made every reasonable effort to include all candidate competitors. These alternatives constitute algorithms specifically designed for MapReduce architectures (i.e., SFO), standard FS algorithms using parallel implementations of the conditional independence tests (i.e., UFS and FBS) and ITFS. The only Lasso implementation for Spark available in the Spark MLlib library (Meng et al. 2016) (a) is for continuous targets, and thus is not suitable for binary classification tasks, and (b) required tuning of 5 hyper-parameters; as no procedure has been suggested by the authors for their tuning, it was excluded from the comparative evaluation. Additionally, for the comparative evaluation, final predictive models were build using the selected features by each algorithm using:

1. The logistic regression implementation in the Spark machine learning library MLlib, hereafter denoted as *SparkLR*
2. The logistic regression model stemming from combining local logistic models using our own implementation (ee Sect. 3.5), hereafter denoted as *CombLR*

The reason for including both types of modeling is because we noticed that SparkLR often fails to converge and produces models that are worse than the trivial classifier classifying to the most common class. This last comparison provides evidence that not only local *p*-values can be combined using meta-analysis techniques, but also model coefficients and other estimated quantities.

### Experimental setup

For the scalability experiments of PFBP (Sect. 9.2) and the proof-of-concept application on SNP data (Sect. 9.4) we used a cluster with 5 machines, 1 acting as a master and 4 as workers, connected to a 1 Gigabit network, with each machine having 2 Intel Xeon E5-2630 v3 CPUs with 8 physical cores each and 256 GB of RAM (a total of 64 cores and 1 TB of RAM). For the comparative evaluation of PFBP with other feature selection algorithms we used a cluster with 5 workers, connected to a 14 Gigabit network, with each machine having 4 Intel Xeon E5-4650 v2 CPUs with a total of 80 cores and 400 GB of RAM (a total of 320 cores and 1.6 TB of RAM). In all cases, 2 cores per worker were left out for other tasks (e.g. the operating system). The first cluster is running Spark 2.1.0 while the second is running Spark 2.0.2, and both are using the HDFS file system. All algorithms were implemented in Java 1.7 and Scala 2.11.

The significance level $\alpha$ was set to 0.01 for all algorithms,^15^ and PFBP was executed with 2 Runs. For the bootstrap tests used by PFBP, we used the default parameter values as described in Sect. 4.2. For each feature selection method we produced two predictive models: (a) using the approach described in Sect. 3.5 that combines multiple locally learned models into a single one, and (b) using the logistic regression implementation in the Spark machine learning library MLlib (Meng et al. 2016). Parameter values related to the number of Group Samples, Sample Sets and Feature Sets were determined using the STD rule, and by setting the maximum number of variables to select to $maxVars$ (the exact value is given for each specific experiment later); see Sect. 5 for more details. We note that, none of the experiments required a physical partitioning to Feature Sets, and thus each Block contains all features (i.e., the number of Features Sets nf is 1).

### Scalability of PFBP with sample size, feature size and number of workers

We investigated the scalability of PFBP on dense synthetic datasets in terms of sample size, variable size and number of workers used. The data were sampled from randomly generated Bayesian networks, which are probabilistic models that can encode complicated dependency structures among features. Such *simulated data contain not only features necessary for optimal prediction* [*strongly relevant in the terminology of* John et al. (1994)] *and irrelevant, but also redundant features* [*weakly relevant* (John et al. 1994)]. This is a novelty in the Big Data FS literature as far as we know, making the synthetic tasks more realistic. A detailed description of the data and network generating procedures is given in “Appendix B”.

For each experimental setting, we generated 5 Bayesian networks, and sampled one dataset from each. The connectivity parameter *C* was set to 10 (i.e., the average degree of each node), the class distribution of *T* was 50/50, and the variance of the error term was set to 1. To investigate scalability in terms of sample size, we fixed the number of features to 1000 and varied the sample size from 2 to 10 M. Scalability in terms of feature size was evaluated on datasets with 100K samples and varying the feature size from 20 to 100 K, all of which also included the optimal feature set (i.e. the Markov blanket of *T*). Finally, scalability in terms of number of workers was investigated on datasets with 10 K variables and 1M samples. The maximum number of variables $maxVars$ to select was set to 50.

**Fig. 3:**
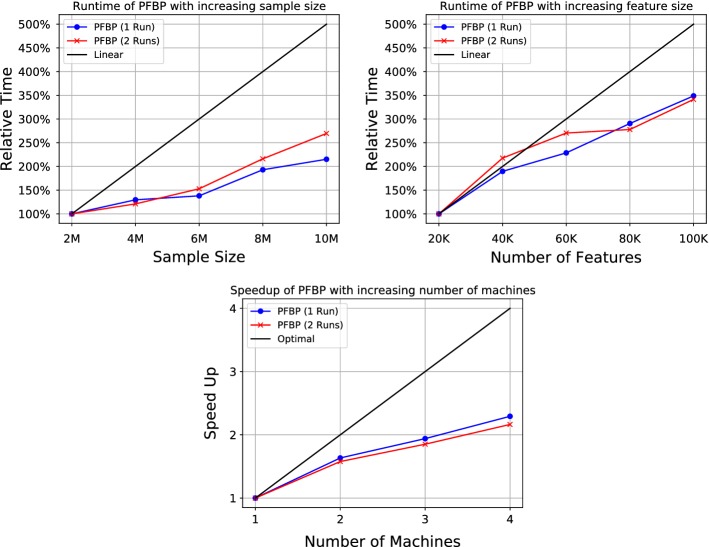
Scalability of PFBP with increasing sample size (top left), feature size (top right) and number of machines (bottom). Time and speed-up were computed relatively to the first point on the x-axis, for the same number of Runs. PFBP improves super-linearly with sample size, linearly with feature size and running time is reduced linearly with increasing number of machines. The results are similar for PFBP with 1 run and 2 runs

The results are summarized in Fig. 3. On the top row we show the relative runtime of PFBP with varying sample size (left) and number of variables (right), respectively. The bottom figure shows the speed-up achieved with varying the number of workers. Relative time and speed up are computed with respect to the lowest point on the x-axis. We can clearly see that: (Top Left) PFBP improves super-linearly with sample size; in other words, feeding twice the number of rows to the algorithm requires less than double the time. This characteristic can be attributed to the Early Stopping and Early Return heuristics. (Top Right) PFBP scales linearly with number of features due to the Early Dropping heuristic and (Bottom) PFBP is able to utilize the allocated machines, although the speed-up factor does not reach the theoretical optimum. The reason for this is that the Early Stopping heuristic quickly prunes many features from consideration after processing the first Group sample, reducing parallelization in subsequent Groups as only few features remain Alive.

**Table 2:** Binary classification datasets used in the comparative evaluation

Name	#Samples	#Variables	Non-zeros per row
SUSY	5,000,000	18	17.79
HIGGS	11,000,000	28	25.79
covtype.binary	581,012	54	11.88
epsilon	500,000	2000	2000.00
rcv1.binary	697,641	47,236	73.15
avazu-app	14,596,137	1,000,000	15.00
avazu-site	25,832,830	1,000,000	15.00
criteo	45,840,617	1,000,000	39.00
news20.binary	19,996	1,355,191	454.99
url	2,396,130	3,231,961	115.63
webspam	350,000	16,609,143	3727.71
kdd2010a	8,407,752	20,216,830	36.35
kdd2010b	19,264,097	29,890,095	29.40

### Comparative evaluation of PFBP on real datasets

We evaluated the PFBP algorithm on 13 binary classification datasets, collected from the LIBSVM dataset repository,^16^ with the constraint that each dataset contains at least 500K samples or variables. A summary of the datasets, shown in order of increasing variable size, is shown in Table 2. The first two columns show the total number of samples and variables of each dataset, while the last column shows the average number of non-zero elements of each sample. The maximum number of non-zero elements equals the total number of variables. Except for the first four datasets, all other datasets are extremely sparse.

All algorithms were compared in terms of classification accuracy and running time. To estimate the classification accuracy, 10% of the training instances were randomly selected and kept out. The remaining 90% were used by each algorithm to select a set of features and to train a logistic regression model using those features. The maximum number of features to select was set to 50. We note that, for PFBP, the backward phases and the second phase were only executed if the algorithm terminated (i.e., the remaining variables were empty) before the variable limit was reached. This was done because PFBP would not have terminated otherwise (i.e., the first phase would still have variables to consider), and thus would not have executed the extra phases. A timeout limit of 12 h was used for each algorithm. In case an algorithm did not terminate within the time limit, the number of features selected up to that point are reported. If no feature was selected, the accuracy was set to N/A (not available). For PFBP, we used the data partitioning strategy described in Sect. 5. For the remaining methods, the number of partitions was set to 4 times the total number of Spark tasks. We ran 6 Spark tasks, each one using 13 cores, on each of the 5 workers. Thus, the total number of partitions was set to 120.

#### Comparison of PBFP with forward selection based methods

We compared PFBP to 3 forward selection based algorithms: (i) Single Feature Optimization (SFO) (Singh et al. 2009), (ii) Forward–Backward Selection (FBS), and (iii) Univariate Feature Selection (UFS). UFS and FBS were implemented using a parallelized implementation of standard binary logistic regression for Big Data provided in the Spark MLLib (Meng et al. 2016).

**Table 3:** The table shows the total running time for each algorithm and dataset

Dataset	Running time (HH:MM)			
	PFBP	SFO	UFS	FBS
SUSY	**00:01**	00:09	00:02	00:40
HIGGS	**00:02**	00:16	00:03	01:59
covtype	00:09	01:05	**00:04**	05:17
epsilon	**00:02**	02:43	00:49	12:00*
rcv1	**00:15**	12:00*	12:00*	12:00*
avazu-app	**04:23**	12:00*	12:00*	12:00*
avazu-site	**05:43**	12:00*	12:00*	12:00*
criteo	**03:15**	12:00*	12:00*	12:00*
news20	**00:44**	12:00*	12:00*	12:00*
url	**01:48**	12:00*	12:00*	12:00*
webspam	**06:13**	12:00*	12:00*	12:00*
kdd2010a	**06:37**	12:00*	12:00*	12:00*
kdd2010b	**10:34**	12:00*	12:00*	12:00*

The fastest algorithms are shown in bold, while algorithms that timed out are indicated with an asterisk. PFBP significantly outperforms all competitors in terms of running time, and is the only algorithm that is able to terminate for all datasets within the given time limit of 12 h. Furthermore, except for 2 cases (FBS on the epsilon dataset and SFO on the rcv1 dataset; see Table 4), none of the competing algorithms were able to select a single variable within 12 h

**Table 4:** The table shows the number of selected variables and the classification accuracy of forward-selection based algorithms on all datasets

Dataset	Classification accuracy (%)									#Selected Variables			
	CombLR				SparkLR				Trivial	PFBP	SFO	UFS	FBS
	PFBP	SFO	UFS	FBS	PFBP	SFO	UFS	FBS					
SUSY	78.91	78.91	78.90	**78.91**	78.59	78.59	**78.61**	78.59	54.22	12	14	18	13
HIGGS	64.26	**64.27**	64.27	**64.27**	64.12	64.15	**64.16**	64.15	53.06	16	18	28	18
covtype	**75.16**	*71*.*02*	*57*.*77*	*57*.*59*	75.80	75.74	**76.00**	73.57	50.78	34	44	50	48
epsilon	**86.05**	85.84	80.08	76.39	**86.07**	85.82	80.02	76.33	51.05	50	50	50	10
rcv1	**91.46**	64.86	N/A	N/A	**91.28**	64.86	N/A	N/A	52.71	50	1	0	0
avazu-app	88.16	N/A	N/A	N/A	*87*.*80*	N/A	N/A	N/A	88.12	50	0	0	0
avazu-site	80.48	N/A	N/A	N/A	*80*.*07*	N/A	N/A	N/A	80.14	50	0	0	0
criteo	76.43	N/A	N/A	N/A	76.37	N/A	N/A	N/A	74.41	50	0	0	0
news20	85.15	N/A	N/A	N/A	*83*.*19*	N/A	N/A	N/A	51.47	50	0	0	0
url	96.93	N/A	N/A	N/A	97.13	N/A	N/A	N/A	67.11	50	0	0	0
webspam	98.08	N/A	N/A	N/A	98.03	N/A	N/A	N/A	60.42	50	0	0	0
kdd2010a	86.12	N/A	N/A	N/A	*57*.*11*	N/A	N/A	N/A	85.33	50	0	0	0
kdd2010b	86.16	N/A	N/A	N/A	*44*.*18*	N/A	N/A	N/A	86.09	50	0	0	0

Classification accuracy is obtained by combining models CombLR (see Sect. 3.5) as well as using the default MLlib logistic regression implementation, SparkLR. Bold numbers show the best performing method for a given classifier, while numbers shown in italic indicate that there is a significant difference ($>1\%$) between the predictive performance obtained using the classifiers, or that the classifier performs worse than the trivial classifier. Overall, all feature selection methods perform similarly, with PFBP and SFO typically having the best predictive performance. PFBP achieves the better or on par performance by selecting fewer variables than its competitors. Furthermore, in most cases, combining models CombLR works as well or better than the logistic regression of MLlib, SparkLR

Table 3 shows the running times of the algorithms (rounded up to the closest minute), and Table 4 show the classification accuracy and the number of selected variables. We included the results of the trivial classifier, which assigns each sample to the most frequent class, and thus attains an accuracy equal to the frequency of the most common class.

*It can be seen that PFBP outperforms all competing methods in terms of running time.* SFO, UFS, and FBS only terminate selecting at least 1 feature in the smallest, first 4 datasets. UFS and FBS reach the timeout limit and do not select a single feature even for the moderately sized rcv1 dataset, which contains only 47K variables and 698K samples, while SFO is able to select only a single feature in 12 h.^17^ PFBP is able to terminate for all datasets within 12 h, taking a maximum of 10.5 h for the kdd2010b dataset which contains 19M samples and 30M variables.

*In terms of predictive performance, PFBP always produces the best or an equally predictive model*. When a competitor produces a better model the difference is in order of 0.01% of accuracy. Some larger differences are observed only when the final model is fit with the SparkLR. *In terms of the number of features, PFBP always selects the lowest number of features* to achieve the same or better performance. An exception is when FBS timed-out for the epsilon dataset selecting 10 features versus 50 for PFBP; however, the lower number of features comes with a significant drop in performance of about 10% of accuracy.

#### Comparison of PBFP with information theoretic feature selection methods

Next, we compare PFBP to three algorithms of the family of information theoretic feature selection (ITFS) methods (Brown et al. 2012): the Minimum-Redundancy Maximum-Relevance (MRMR) algorithm (Peng et al. 2005), the Joint Mutual Information (JMI) algorithm (Yang and Moody 2000) and the Conditional Mutual Information Maximization algorithm (Fleuret 2004). Those methods were chosen as they have been shown to be perform well compared to several other members of the ITFS family (Brown et al. 2012). For all of the above algorithms, we used existing Spark-based implementations^18^ (Ramrez-Gallego et al. 2017).

As information-theoretic feature selection methods require discrete data, we performed a simple discretization method on all sparse datasets (i.e., except for SUSY, HIGGS, covtype and epsilon), by setting the value to 0 if the original value was 0, and to 1 otherwise. Thus, a 0 or 1 indicates the absence or presence of a value respectively. Although this discretization method may be sub-optimal, it still allows for a fair comparison between all methods, as they are all executed on the same data. Furthermore, discretization to more than 2 values would put PFBP at a disadvantage, as the independence tests based on logistic regression models would need to fit models for more parameters. Specifically, if a discrete variable takes *K* values, logistic regression would need to fit $K-1$ coefficients. As we will see below, this discretization method does not significantly affect the results in terms of classification accuracy, justifying its use in this case. On the contrary, in some cases the produced models have a higher accuracy than the ones obtained from the original data.

**Table 5:** The table shows the total running time of each algorithm on all discretized datasets

Dataset	Running time (HH:MM)			
	PFBP	MRMR	JMI	CMIM
rcv1	00:13	**00:06**	00:06	00:07
avazu-app	06:00	00:16	**00:16**	00:16
avazu-site	06:02	00:29	**00:25**	00:32
criteo	03:56	**01:15**	01:36	01:23
news20	01:02	00:03	**00:03**	00:04
url	02:20	00:15	**00:14**	00:16
webspam	02:00	**00:53**	01:04	00:58
kdd2010a	07:03	00:25	**00:22**	00:23
kdd2010b	08:49	00:43	**00:40**	00:48

The fastest algorithm for each dataset is shown in bold. ITFS methods significantly outperform PFBP in terms of running time, being almost 23 times faster than PFBP (for the avazu-app dataset)

**Table 6:** The table shows the classification accuracy % for each algorithm and dataset

Dataset	Classification accuracy (%)								
	CombLR				SparkLR				Trivial
	PFBP	MRMR	JMI	CMIM	PFBP	MRMR	JMI	CMIM	
rcv1	**90.64**	86.14	85.64	85.60	**90.87**	86.35	86.20	85.63	52.71
avazu-app	**88.19**	88.14	*87*.*89*	88.15	*87*.*45*	*87*.*54*	*88*.*11*	*88*.*08*	88.12
avazu-site	80.48	**80.50**	*79*.*74*	*79*.*79*	*79*.*77*	80.33	**80.42**	**80.42**	80.14
criteo	**76.39**	76.19	75.98	75.91	**76.32**	75.92	75.98	75.91	74.41
news20	**86.03**	81.22	79.79	79.27	***83***.***16***	77.43	77.12	78.30	51.47
url	**96.80**	94.87	95.79	95.07	**96.98**	95.77	95.36	95.70	67.11
webspam	**98.22**	94.30	94.62	93.92	**98.22**	89.52	91.23	90.88	60.42
kdd2010a	86.17	**86.27**	86.15	86.10	*59*.*15*	*38*.*89*	*30*.*20*	*29*.*58*	85.33
kdd2010b	**86.10**	*86*.*08*	*86*.*07*	*86*.*07*	*43*.*17*	*37*.*98*	*38*.*22*	*37*.*49*	86.09

Classification accuracy is obtained by combining models (see Sect. 3.5) as well as using the default MLlib logistic regression implementation. Bold numbers show the best performing method for a given classifier, while numbers shown in italic indicate that the classifier performs worse than the trivial classifier. In most cases, PFBP produces better methods, often significantly so, having a higher accuracy of up to 5–9% on the rcv1, news20 and webspam datasets. As before, in most cases, combining models works as good or better than the implementation in MLlib

The results are summarized in Tables 5 and 6. They show the running time in hours and minutes (rounded up to the closest minute) and the classification accuracy for each algorithm. The number of selected variables is not shown, as all algorithms terminated within the time-limit of 12 h and selected 50 variables.

As expected, *regarding running time, ITFS methods clearly outperform PFBP, being about 2–23 times faster than PFBP*. As explained in the discussion in Sect. 8.3, this is because PFBP treats data as dense and is not specific or optimized for discrete data. In addition, PFBP’s current implementation is based on fitting logistic regression models that require iterative techniques, while ITFS methods only require the counts in the contingency tables of the joint distributions.

*In terms of classification accuracy, PFBP outperforms ITFS methods in most cases.* In some cases (rcv1, news20, webspam) the accuracy difference is more than 4%; when PFBP does not produce the best model, the accuracy difference is less than 0.1% . Thus, overall, if the goal is predictive accuracy, PFBP should be preferred over ITFS methods.

**Table 7:** Difference in classification accuracy between models obtained using CombLR and SparkLR across all experiments

Dataset	Continuous data				Discretized data			
	PFBP	SFO	UFS	FBS	PFBP	MRMR	JMI	CMIM
SUSY	0.32	0.32	0.29	0.32	N/A	N/A	N/A	N/A
HIGGS	0.14	0.12	0.11	0.12	N/A	N/A	N/A	N/A
covtype	$-$ 0.64	$-$4.72	$-$ 18.23	$-$15.98	N/A	N/A	N/A	N/A
epsilon	$-$ 0.02	0.02	0.06	0.06	N/A	N/A	N/A	N/A
rcv1	0.18	0.00	N/A	N/A	$-$ 0.21	$-$0.21	$-$ 0.76	$-$0.03
avazu-app	0.36	N/A	N/A	N/A	0.74	0.70	$-$ 0.22	0.07
avazu-site	0.41	N/A	N/A	N/A	0.71	0.17	$-$ 0.68	$-$0.63
criteo	0.06	N/A	N/A	N/A	0.07	0.27	0.00	0.00
news20	1.96	N/A	N/A	N/A	2.87	3.79	2.67	0.97
url	$-$ 0.20	N/A	N/A	N/A	$-$ 0.18	$-$0.90	0.43	$-$ 0.63
webspam	0.05	N/A	N/A	N/A	0.00	4.78	3.39	3.04
kdd2010a	29.01	N/A	N/A	N/A	27.02	47.38	55.95	56.52
kdd2010b	41.98	N/A	N/A	N/A	42.93	48.10	47.85	48.58

Positive values indicate that CombLR performs better. In the continuous data from the comparison between PFBP, SFO, UFS and FBS, N/A values correspond to cases where the algorithm did not terminate. For the discretized data, N/A values correspond to cases where the experiment was not performed. In all cases, PFBP using CombLR produces models with similar or better performance than SparkLR

#### Comparison of CombLR and SparkLR

We proceed by comparing the performance obtained using different logistic regression classifiers, namely SparkLR and CombLR. We remind the reader that CombLR comes at no additional computational overhead by combining the coefficients of local models already produced by PFBP for feature selection purposes. SparkLR in contrast, fits a global LR model, with a corresponding computational overhead. Table 7 shows the difference in accuracy obtained by CombLR and SparkLR over all experiments, with positive values corresponding to cases where CombLR outperforms SparkLR.

For the comparison between PFBP, SFO, UFS and FBS on the original datasets, CombLR slightly outperforms SparkLR on most datasets. There are however a few cases where large differences between both classifiers can be seen. For covtype, CombLR results in a large performance drop for SFO, UFS and FBS. However, the performance of PFBP is similar, regardless of the method used for producing the classifier. For PFBP, for kdd2010a and kdd2010b, SparkLR completely fails, achieving an accuracy lower than the one obtained by the trivial classifier (see Table 4). Regarding the comparison of PFBP with information-theoretic methods on the discretized data, we observe again a qualitatively similar behavior as in the previous experiments. The problematic datasets seem to be news20, webspam, kdd2010a and kdd2010b. As before, there are cases where SparkLR fails to produce a model better than the trivial classifier, whereas CombLR is more robust overall. The difference may exceed 50% of accuracy! When CombLR fails to beat the trivial classifier the accuracy difference is less than 0.5% of accuracy; this case never happen for the PFBP algorithm, arguably due to a better selection of features with less collinearities (deterministic relations). In any case, *it is encouraging that in all cases, PFBP using CombLR produces models on par or better than SparkLR*.

Unfortunately, we were not able to determine the cases where SparkLR fails. Specifically, we tried to (a) vary the number of partitions used, and (b) relax the stopping conditions used by the model fitting procedures (increasing number of iterations and reducing tolerance of the termination criterion), but neither of those made any difference.

### Proof-of-concept application on genetic SNP data

Single Nucleotide Polymorphisms (SNPs),^19^ the most common type of genetic variation, are variations of a single nucleotide at specific loci in the genome of a species. The Single Nucleotide Polymorphism Database (dbSNP) (build 150) (Sherry et al. 2001) now lists 324 million different variants found in sequenced human genomes.^20^ In several human studies, SNPs have been associated with genetic diseases or predisposition to disease or other phenotypic traits. As of 2018-07-17, the GWAS Catalog^21^ contains 3471 publications and 65793 unique SNP-trait associations. Large scale studies under way [e.g., Precision Medicine Initiative (Collins and Varmus 2015)] intend to collect SNP data in large population cohorts, as well as other medical, clinical and lifestyle data. The resulting matrices may end up with millions of rows, one for each individual, and variables (SNP or some other measured quantity). Thus, we believe that investigating the behavior of newly proposed Big Data FS algorithms on such data is worthwhile. A proof-of-concept application of PFBP is presented next.

#### SNP data generation and setup

We simulated genotype data containing 500,000 individuals (samples) and 592,555 SNP genotypes (variables), following the procedure described in Canela-Xandri et al. (2015). As SNP data are dense, they require approximately 2.16 TB of memory, and thus are more challenging to analyze than sparse data, such as the ones used in the previous set of experiments. The data were simulated with the HAPGEN 2 software (Chang et al. 2015) from the Hapmap 2 (release 22) CEU population (Consortium 2015). A more detailed description of the data generation procedure is given in “Appendix C”.

We used $M = 100$ randomly selected SNPs to generate a binary phenotype (outcome), as described in Canela-Xandri et al. (2015) (see also “Appendix C”). The optimal accuracy using all 100 SNPs is 81.42%. Ideally and given enough samples, any feature selection method should select those 100 SNPs and achieve an accuracy around 81.42%. Due to linkage disequilibrium however, many neighboring SNPs are highly correlated (collinear) and as a consequence offer similar predictive information about the outcome and are informationally equivalent. Therefore, a high accuracy can be achieved even with SNPs other than the 100 used to simulated the outcome.

We used 95% of the samples as a training set, and 5% as a test set for performance estimation. We set a timeout limit of 15 h, and used the same setup as used in previous experiments, with the exception that the maximum number of variables to select was set to 100.

#### Repartitioning to reduce memory requirements

For big, dense data such as the SNP data considered in this experiment which require over 2 TB of memory, a direct application of PFBP as used in other experiments is possible, but may be unnecessarily slow. We found that for such problems, it makes sense to repartition the data at some point, if enough variables have been removed by the Early Dropping heuristic. Repartitioning and discarding dropped variables reduces storage requirements, and may offer a significant speed boost. It is an expensive operation however, and should only be used in special situations. For the SNP data, after the first iteration only about a third of the variables remained, reducing the memory requirements to less than 1 TB, and thus most (if not all) of the data blocks were able to fit in memory. In this case repartitioning makes sense, as its benefits far outweigh the computational overhead.

#### Results on the SNP data

PFBP was able to select 84 features in 15 h, using a total of 960 core hours. It achieved an accuracy of 77.62%, which is over 95% of the theoretical optimal accuracy. The results are very encouraging; in comparison the DISSECT software (Canela-Xandri et al. 2015) took 4 h on 8400 cores (that is, 33,600 core hours) and using 16 TB of memory to fit a mixed linear model on similar data, and to achieve an accuracy around 86% of the theoretical maximum. The two experiments are not directly comparable because (a) the outcome in our case is binary instead of continuous requiring logistic regression instead of linear regression models favoring DISSECT in terms of computational time, (b) the scenarios simulated in Canela-Xandri et al. (2015) had larger Markov blankets (1000 and 10000 instead of 100) favoring PFBP (although, their results are invariant to the size of the Markov blanket). Nevertheless, the reported results are still indicative of the efficiency of PFBP on SNP Big Data.

**Fig. 4:**
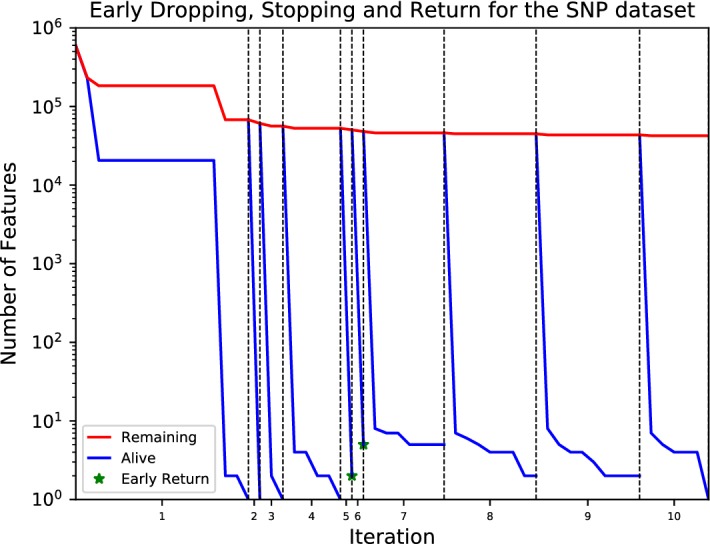
The effects of the early pruning heuristics is shown for the first 10 forward iterations on the SNP data. The y-axis shows the number of variables on a logarithmic scale. The width of each iteration is proportional to the number of groups processed. The Early Dropping heuristic is able to quickly discard many features, reducing them by about an order of magnitude. Early stopping filters out most variables after processing the first group, and early return is applied two times

Figure 4 shows the effects of the heuristics used by PFBP for the first 10 iterations. The y-axis shows the number of Remaining and Alive features on a logarithmic scale. The x-axis shows the current iteration, and the width is proportional to the total number of Groups processed in that iteration. We observe that (a) Early Dropping discards many features in the first iteration, reducing the number of Remaining features by about an order of magnitude, (b) in most iterations, Early Stopping is able to reduce the number of Alive features to around 10 after processing the first Group, (c) Early Return is applied 2 times, ending the Iteration and selecting the top feature after processing a single Group.

**Fig. 5:**
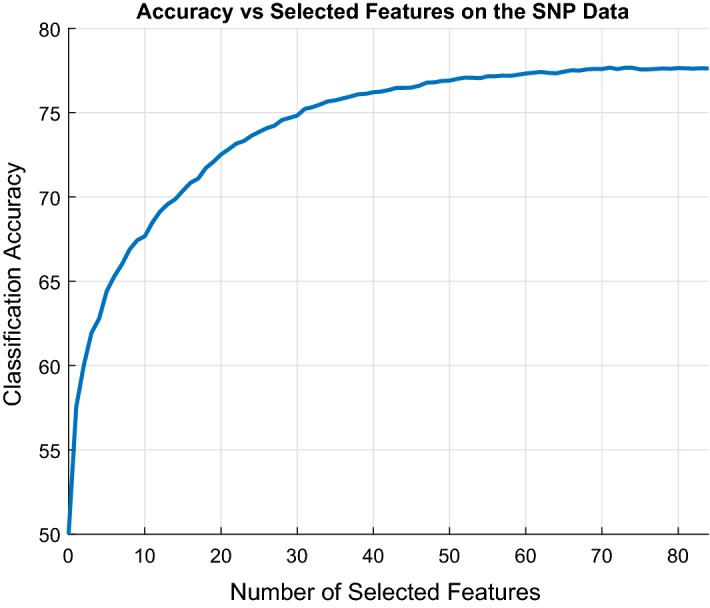
The figure shows how the accuracy of PFBP on the SNP data increases as it selects more features. The models are produced by PFBP at each iteration with minimal computational overhead. In the first few iteration, accuracy increases sharply, while in the later iterations a plateau is reached, reaching a value of 77.59% with 70 features, with the maximum being 77.62% with 84 features. This could be used as a criterion to stop feature selection early

Finally, by combining the intermediate logistic regression models at each Iteration using CombLR, we computed the accuracy at each iteration of PFBP with no additional overhead. This provides an insight regarding its predictive performance behavior with increasing number of selected features. As before, the accuracy is computed on the 5% of the data that were kept out as a test set. Such information could be used to decide early whether a sufficient number of features has been selected, and to stop computation if the accuracy reaches a plateau. This is often the case, as most important features are typically selected during the first few iterations. This task can be performed using PFBP with minimal computational overhead, as the local models required to approximate a full global model (see Sect. 3.5) are already available. The results are shown in Fig. 5. As expected, the largest increase in accuracy is obtained after selecting the first few features, reaching an accuracy of 75% even after selecting only 30 features. In addition, after selecting about 70 features, the accuracy increases only marginally afterwards, increasing from 77.59% with 70 features to 77.62% with 84. Thus, computation could be stopped after 70 features have been selected, and still attain almost the same accuracy.

### Summary and discussion of experimental results

Overall, the experiments indicate that PFBP scales superlinearly with the available sample size, and linearly with the number of features and available workers. Compared with other algorithms in its class, namely forward selection-based algorithms with map-reduce implementations, under the same conditions (type of test and predictive model), PFBP dominates the alternatives (UFS, FBS, SFO) in terms of execution time, number of selected features, and predictive performance. Against information-theoretic variants specialized for discrete and sparse data with available map-reduce implementations, PFBP performs worse in terms of running time, however, it is still applicable and practical to apply to large datasets. However, PFBP dominates the information-theoretic variants in terms of predicting performance. Furthermore, as a side product of the experiments, we compared two logistic regression algorithms, namely SparkLR that is available in MLlib and fits in a parallelized fashion a global logistic regression model, and CombLR that combines the coefficients of local logistic regression models. CombLR always converges, providing on average more predictive models than SparkLR, and it is considerably more efficient than SparkLR even when computed from scratch and not during PFBD. Finally, the proof-of-concept application to SNP data demonstrates that the emergence of Big genetic Data can become amenable to analysis using algorithms such as PFBP. A detailed trace of the computational experiment shows the effectiveness of the Early Stop, Drop and Return heuristics of PFBP: (a) after the first few iterations the Remaining features are reduced by 1–2 orders of magnitude. (b) The number of Alive features drops exponentially as more groups are processed. The trace visualizes PFBP’s scalability properties.

## Discussion and conclusions

We present a novel algorithm for *feature selection* (FS) in Big Data settings called Parallel, Forward–Backward with Pruning (PFBP). PFBP is a general algorithm for any type of data and outcome, by equipping it with an appropriate conditional independence test. It works on both dense and sparse data. PFBP can scale to millions of predictive quantities (i.e., features, variables) and millions of training instances (samples). The Parallel, Forward–Backward with Pruning (PFBP) enables *computations that can be performed in a massively parallel way* by partitioning data both *horizontally* (over samples) and *vertically* (over features) and using meta-analysis techniques to combine results of local computations. Similar meta-analysis tricks can combine local logistic regression coefficients to global models with excellent results in practice against the global logistic regression models produced by MLlib. PFBP is equipped with *heuristics that can quickly and safely drop from consideration some of the redundant and irrelevant features* to significantly speed up computations. The heuristics are inspired by causal models and provide theoretical guarantees of correctness in distributions faithful to causal models (Bayesian networks or maximal ancestral graphs). Bootstrapping testing allows PFBP to determine whether enough samples have been seen to safely apply the heuristics and forgo computations on the remaining samples. Our empirical analysis confirms that, PFBP exhibits a super-linear speedup with increasing sample size and a linear scalability with respect to the number of features and processing cores. A comparative evaluation shows that PFBP dominates other alternative map-reduce algorithms in its class in terms of computational performance, number of selected features, and predictive performance. Against information theoretic algorithms, specialized for sparse, discrete data it is slower, but returns models with higher predictive performance. PFBP was tested on high dimensional SNP data of about 500K demonstrating its applicability to dense, genomic data. A limitation to address in the future is to equip the algorithm with a principled criterion for the determining the number of selected features. Other directions to improve include exploiting the sparsity of the data, implementing run-time re-partitioning when deemed beneficial, and implementing tests in GPUs to name a few.

## Figures and Tables

**Fig. 6:**
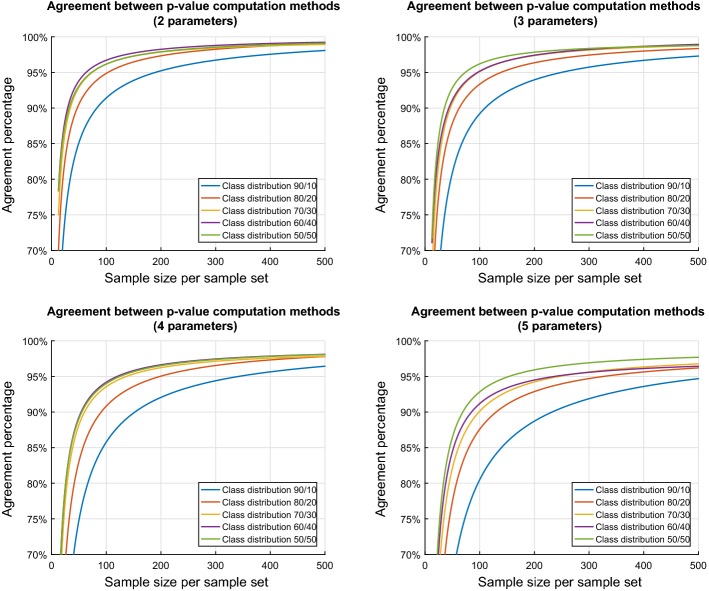
The percentage of agreement is shown, which corresponds to how often combining local *p*-values and computing the *p*-value on all samples leads to the same decision. The *y*-axis shows how the sample size per sample set affects the agreement percentage. Both methods tend to agree asymptotically for various class distributions and conditioning set sizes

## A: Accuracy of *p*-value combination using meta-analysis and evaluation of the STD rule

We evaluated the ability of the proposed *p*-value computation method (combination of local *p*-values using Fisher’s combined probability test) in identifying the same variable to select as when global *p*-values are used. We performed a computational experiment on simulated data to investigate the effect of the total sample size and number of data Blocks on the accuracy of the proposed approach. Furthermore, we compare the STD and EPV rules for setting the minimum number of samples in each Data Block. The EPV rule computes the sample size per Sample Group as $s = df \cdot c / \min (p_0,p_1)$, while STD uses $s = df \cdot c / \sqrt{p_0 \cdot p_1}$, where *df* is set to the maximum number of degrees of freedom (see 5.1 for more details), *c* is a positive constant (which may take different values for each rule), and $p_0$ and $p_1$ are the frequencies of the negative and positive class respectively.

### A.1: Data generation

To generate data with complex correlation structures, we chose to generate data from simulated Bayesian networks. All variables are continuous Gaussian and are linear functions of their parents. The target variable is binary, and the log-odds ratio is a linear function of its parents. The procedure is described in detail in “Appendix B”.

We used the following parameters to generate Bayesian networks and data from those networks: (a) the number of variables was set to 101 (100 variables plus the outcome *T*), (b) the connectivity parameter was set to 10 (i.e., the average degree of each node), (c) the frequency of the most frequent class of *T* was set to $\{50, 60, 70, 80, 90\%\}$ and (d) the standard deviation of the error terms was set to $\{0.01, 0.1, 1\}$. In total this results in 15 possible Bayesian network configurations. Note that, the connectivity is relatively high and the standard deviation of the error terms is relatively low so that all variables are highly correlated, increasing the difficulty of the problem of selecting the best variable. For each such parameter combination we generated 250 Bayesian networks, resulting in a total of $250 \times 15 = 3750$ networks. Next, we generated datasets with different sample sizes, by varying the sample size from $10^{2.5}$ to $10^4$ in increments of 0.1 of the exponent, leading to 16 different sample sizes. Overall this resulted in 60,000 datasets.

### A.2: Simulation results: combined *p*-values versus global *p*-values

We performed conditional independence tests on all generated datasets to simulate a forward Iteration using *p*-values from global tests (i.e., using all data) and from combined *p*-values using Fisher’s method. We varied the following parameters: (a) the number of conditioning variables, which was set to 0, 1, 2 or 3, and (b) the number of Sample Sets each dataset was split to, which ranged from 1 (no split) to 25 with increments of 1 (a total of 25 cases). This allows us to investigate the effect of the total number of combined local *p*-values. The simulation of a forward Iteration was performed for each dataset and conditioning size *k* as follows: (a) *k* variables were randomly selected from the Markov blanket of *T* (simulating that *k* variables have already been selected), (b) the global conditional independence test was performed between *T* and the remaining variables over all samples (i.e. number of sample sets equals 1), (c) the same test was performed on all Sample Sets resulting by splitting the data randomly to *m* equally-sized sample sets (*m* ranging from 2 to 25) and combining the *p*-values using Fisher’s combined probability test.

We compute the percentage of agreement between both methods, that is, how often both methods select the same variable. This is computed as the proportion of times both methods agreed on the 250 repetitions, leading to one value for each of the 15 Bayesian network configurations, each sample size, conditioning set size and number of Sample Sets. Thus, in total we have $15 \times 16 \times 4 \times 24$ = 23,040 such values. The results are summarized in Fig. 6. There are 4 figures, one for each different conditioning size, and each figure contains 5 curves, one for each class distribution of *T*. Each such curve summarizes the results over all error variances, sample sizes and number of Sample Sets (that is, $3 \times 16 \times 24$ = 1152 points). Note that the number of parameters of the largest model is always the conditioning size plus 2, as the model also includes the variable that is tested for (conditional) independence with *T* and the intercept. The *x*-axis shows the sample size per Sample Set, which is computed as the sample size divided by the number of Sample Sets. We only show the results up to 500 samples per Sample Set; the agreement percentage approaches $100\%$ in all cases with increasing sample size, reaching at least $99\%$ with 5000 samples per Sample Set. The *y*-axis shows how often both methods lead to the same decision. To avoid cluttering, we computed the curves by fitting a power regression model $y = \alpha \cdot x^\beta + c$. We found that this model is appropriate, as it has $R^2$ values between 0.75 and 0.95. We conclude the following: *(a) both approaches tend to make the same decision with increasing sample size, (b) the sample size per Sample Set required depends on the number of parameters and the class distribution, and increases with increasing number of parameters and class imbalance*.

### A.3: Simulation results: STD versus EPV for determining the required sample size

We propose an alternative rule to EPV, which is computed as $df \cdot c/\sqrt{p_0 \cdot p_1}$. The denominator is the standard deviation of the class distribution, which follows a Bernoulli distribution. We call this the STD rule hereafter. For balanced class distributions the result is identical to the EPV rule, while for skewed distributions the value is always smaller.

To validate the STD rule, we used the results of the previous simulation experiment and computed the value of *c* by solving the equation for *c* and substituting in the values of the class distributions, degrees of freedom and sample size per Sample Set. We kept the values of *c* that correspond to an agreement percentage between 85 and $95\%$ (focusing on an interesting range of high agreement between *p*-value computation methods), and computed their median value for each class distribution, conditioning size *k* and for both rules. Ideally, one would expect *c* to be constant across rows (class distribution) and columns (conditioning size). A constant value of *c* for a rule means that the rule can exactly compute the required sample size to get an agreement percentage around $90\%$. Furthermore, we note that the values of *c* are not comparable between rules, and thus their exact values are not important; what matters is the relative difference between values of *c* for the same rule.

**Table 8:** Median value of *c* to obtain an agreement percentage between 85 and $95\%$

$p_{max}$ | df	EPV Rule				STD Rule			
	2	3	4	5	2	3	4	5
0.5	11.2	7.8	9.0	9.7	11.2	7.8	9.0	9.7
0.6	7.7	7.6	7.4	11.2	9.4	9.3	9.1	13.7
0.7	6.6	5.7	5.9	8.6	10.1	8.8	9.1	13.2
0.8	5.7	5.2	5.7	6.7	11.5	10.5	11.4	13.3
0.9	5.0	4.4	4.2	4.4	14.9	13.2	12.5	13.3

$p_{max}$ corresponds to the proportion of the most frequent class, while df is the degrees of freedom in the largest model. The relative differences for the STD rule are smaller (less than 2 against over 2.5 for the EPV rule), suggesting it is more appropriate. A minimum value of $c = 10$ with the proposed rule is recommended and used in the experiments

The results are shown in Table 8. Although the value of *c* varies across class distributions and degrees of freedom, we can see that the relative differences are smaller the STD rule. Specifically, for EPV *c* ranges from 4.2 to 11.2, the latter being over 2.5 times larger, while for STD it ranges from 7.8 to 14.9, which is less than 2 times larger. This suggests that the STD rule performs better than EPV across various conditioning set sizes and class distributions, at least on the experiments considered here. Furthermore, the results suggest that a value of at least $c = 10$ should be used for STD to get reasonably accurate results. We note that, in practice this value is much higher in most cases for PFBP, as it partitions the samples initially by considering the worst case scenario (i.e., selecting $maxVars$ variables). Thus especially in early Iterations, which are the most crucial ones, PFBP will typically have a sufficient number of samples even with $c = 10$ to select the best variables.

## B: Simulating data from Bayesian networks

To generate data with complex correlation structures, we chose to generate data from Bayesian networks. This is done in three steps: (a) generate a Bayesian network structure $\mathcal {G}$ with *N* nodes (variables) and *M* edges, (b) sample the parameters of $\mathcal {G}$, and (c) sample instances from $\mathcal {G}$. We will next describe the procedures used for each step.

### B.1: Generating the Bayesian network structure

First, we need to specify the number of nodes *N* and the connectivity *C* between those nodes, which implicitly corresponds to some number of edges *M*. The connectivity parameter *C* corresponds to the (average) degree of each variable. Using the connectivity instead of setting the number of edges allows one to easily control the complexity of the network, as *C* directly corresponds to the average number of parents and children of each node. We proceed by showing how the edges were sampled. Let $V_1, \dots , V_N$ be all nodes of $\mathcal {G}$, listed in topological order. To sample the edges of the network we iterate over all pairs of variables $V_i$ and $V_j\,(\hbox {i} < \hbox {j})$, and add an edge from $V_i$ to $V_j$ with probability $C/(N-1)$, ensuring acyclicity of the resulting graph. It can be easily shown that this will result in a network with an average degree of *C*.

### B.2: Generating the Bayesian network parameters

The first step is to pick the variable that corresponds to the outcome variable *T*. We chose to use the node at position $\lceil N/2 \rceil$, as this node has the same number of parents and children on average. For our experiments, we chose *T* to be of binary type and all remaining variables to be of continuous type, but in principle everything stated can be easily adapted to other variable types. In Bayesian networks, the each variable *V* is a function of its parents $\mathbf {Pa}(V)$. The functional form for continuous variables $V_i$ is

$$\begin{aligned} V_i = \beta _0 + \sum _{V_j \in \mathbf {Pa}(V_i)} \beta _j V_j + \epsilon _i \end{aligned}$$

where $\beta _0$ is the intercept, $\beta _j$ is the coefficient of the *j*th parent of $V_i$ and $\epsilon _i$ is its error term. In our case, we set the intercept to 0, as it does not affect the correlation structure. The coefficients $\beta _j$ are sampled uniformly at random from $[-1,-0.1] \cup [0.1,1]$ to avoid coefficients which are close to 0. The error term $\epsilon _i$ follows a normal distribution with 0 mean and $\sigma _i^2$ variance, which was set to the default value of 1 in our experiments, unless stated otherwise. Note that all variables are normally distributed as they are sums of normally distributed variables. For each variable $V_i$ the mean equals zero and the variance equals $\sigma _i^2 + \sum _{V_j \in \mathbf {Pa}(V_i)} \beta _j^2$. The fact that the variance increases may lead to numerical instabilities in practice, especially when generating large networks. Because of that, we standardize each variable to have unit variance by dividing it with its standard deviation, which is the square root of the variance as described above. For the target *T*, its log-odds ratio is again a linear function of its parents, defined as

$$\begin{aligned} \log \left( \frac{P(T = 1)}{1 - P(T = 1)}\right) = \beta _0 + \sum _{V_j \in \mathbf {Pa}(T)} \beta _j V_j + \epsilon _T \end{aligned}$$

As before, the log-odds ratio is standardized to have unit variance.

The value of *T* is set to 1 whenever the log-odds ratio is larger than some threshold *t*, and to 0 otherwise. Setting *t* to 0 results in a 50/50 class distribution of *T*. Other class distributions $p_0/p_1$ can be obtained by simply setting *t* to $N_{0,1}^{-1}(1-p_0)$, where $N_{0,1}^{-1}$ is the standard normal inverse cumulative distribution function. As a final note, the standardization method used above only guarantees that variables that come before *T* in the topological ordering are standard normal variables. As *T* is not normally distributed (nor does it have unit variance), all variables that are direct or indirect functions of *T* are not exactly normally distributed. However, as this neither alters the correctness of the data generation method, nor leads to any other issues, we leave it as is.

### B.3: Sampling data from the generated Bayesian network

To generate a sample, one has to traverse the network in topological order and to compute the value of each variable separately, using the formulas described previously. By construction the network is already in topological order, which is simply given by the index of each variable. To compute the value of a variable one has to compute the sum of its parents (if it has any parents), and to add the error term, which is drawn from a normal distribution.

## C: SNP data generation

To generate the SNP dataset we followed the procedure described in Canela-Xandri et al. (2015). We used the HAPGEN 2 software (Chang et al. 2015) with the Hapmap 2 (release 22) CEU population (Consortium 2015) to simulate 500000 individuals (samples). This population contains 2543887 SNPs, but only 592555 were kept, by filtering out the ones not available in the Illumina Human OmniExpress-12 v1.1 BeadChip.^22^ The final dataset contains 500000 samples and 592,555 SNPs. Each variable takes values in $\{0,1,2\}$, which correspond to the number of reference alleles. Thus, the dataset is dense, and requires approximately 2.16 TB memory (stored as double precision floats). Naturally, fewer bytes can be used to store SNP data as each variable only takes 3 values, but this would require a specialized implementation.

### C.1: Phenotype simulation

Let $s_{ij}$ be the *i*th value of the *j*th SNP $s_j$, and $p_j$ be the reference allele frequency of SNP *j*, that is, $p_j$ is the average value of $s_{ij}$ divided by 2. The standardized value of $s_{ij}$, $z_{ij}$ is defined as

$$\begin{aligned} z_{ij} = \left( s_{ij} - \mu _j\right) / \sigma _j \end{aligned}$$

where $$\mu _j = 2 p_j$$ and $$\sigma _j = \sqrt{2 p_j (1 - p_j)}$$.

The phenotype (outcome) *y* follows an additive genetic model

$$\begin{aligned} y_i = g_i + e_i = \sum _{j=1}^M z_{ij} u_j + e_i \end{aligned}$$

where $y_i$ is the *i*th value of *y*, $g_i$ is the genetic effect, $e_i$ is the noise term, *M* is the number of variables influencing *y*, and $u_j$ is the effect (coefficient) of $z_j$. The coefficients $z_j$ were sampled from a normal distribution with zero mean and unit variance. The error terms $e_i$ follow a normal distribution with zero mean and variance $\sigma _i^2 (1-h^2)/h^2$, where $\sigma _i^2$ is the variance of $g_i$ and $h^2$ corresponds to the trait heritability. Naturally, the larger $h^2$, the more *y* depends on the SNPs. In our case, we chose $M = 100$ and set $h^2 = 0.7$, one of the values used in Canela-Xandri et al. (2015). Finally, to obtain a binary outcome, we set the value of $y_i$ to 1 if it is positive, and to 0 otherwise, resulting in an approximately balanced outcome.

## Footnotes

1 Optimally predictive with respect to an ideal predictor; see Tsamardinos and Aliferis (2003) for a discussion.

2 This definition covers what we call single FS; the problem of multiple FS can be defined as the problem of identifying all minimal and optimally-predictive subsets but it has received much less study in the literature (Statnikov et al. 2013; Lagani et al. 2017).

3 See Zhao et al. (2013), Zhai et al. (2014) and Bolón-Canedo et al. (2015a, b) for the evolution of Big Data dimensionality in various ML datasets.

4 Alternatively, one can combine the test statistics that produce the *p*-values. This is conceptually equivalent, although there may be differences in practice.

5 Alternatively, one can use information criteria such as AIC (Akaike 1973) and BIC (Schwarz 1978), or out-of-sample methods such as cross-validation to evaluate the performance of the current set of selected features; see Kutner et al. (2004) and Weisberg (2005) for more details.

6 For example, the R-package MXM (Lagani et al. 2017) includes asymptotic, permutation-based, and robust tests for nominal, ordinal, continuous, time-course, percentage, count, and censored time-to-event targets.

7 An implicit assumption made here is that the models are correctly specified. If this does not hold, the statistic follows a different distribution (Foutz and Srivastava 1977). There exist methods that handle the more general case (White 1982; Vuong 1989), but this is clearly out of this paper’s scope.

8 Often, the original sample is not considered and thus the estimate is computed by using only the bootstrap samples and dividing by *B*. However, it has been noted (in the context of bootstrap-based hypothesis testing) that one should also consider the original sample statistic [see Section 4.2 in Davison and Hinkley (1997)], which is why we chose to do so too.

9 Some recent algorithms (Statnikov et al. 2013; Lagani et al. 2017) deal with the problem of solution multiplicity in feature selection.

10 Naturally, any method for combining *p*-values can be used instead of Fisher’s method, but we did not further investigate this in this work.

11 One case where this happens is if the covariance matrix of the input data is singular, or close to singular. Note that, due to the nature of the feature selection method which considers one variable at a time, this can happen only if the newly added variable is (almost) collinear with some of the previously selected variables. If this is the case, the variable would not be selected anyway.

12 https://spark-packages.org/package/sramirez/spark-infotheoretic-feature-selection.

13 There are methods that do not fall into this framework, but we will not go into more detail; see Brown et al. (2012) for more details.

14 Recently, there have been efforts for exact algorithms solving the BSS problem using mixed-integer optimization formulations for linear regression (Bertsimas et al. 2016) and logistic regression (Sato et al. 2016).

15 Typical choices for the significance level $\alpha$ are 0.01 and 0.05 (Aliferis et al. 2010). Using lower values of $\alpha$ leads to fewer type I errors (falsely selected variables) but more type II errors (falsely rejected variables). This also depends on the sample size, with more samples typically leading to fewer type II errors, with type I errors not affected by sample size. Therefore, in large sample settings, as the ones considered in our experiments, one should use lower values for $\alpha$ to minimize type I errors. For that reason, we chose $\alpha = 0.01$, although it would make sense to consider even lower values.

16 http://www.csie.ntu.edu.tw/~cjlin/libsvmtools/datasets/.

17 We tried running SFO, UFS and FBS on some of the large datasets using a timeout limit of 2 days, the maximum possible on the cluster we used; however, none of the algorithms were able to select even a single variable.

18 https://github.com/sramirez/spark-infotheoretic-feature-selection.

19 https://ghr.nlm.nih.gov/primer/genomicresearch/snp.

20 https://ncbiinsights.ncbi.nlm.nih.gov/2017/05/08/dbsnps-human-build-150-has-doubled-the-amount-of-refsnp-records/.

21 https://www.ebi.ac.uk/gwas/.
